# Simple Synthesis of Fused 1,3,4-Thiadiazines Through Successive S_N_Ar Processes

**DOI:** 10.3390/ijms27198886

**Published:** 2026-10-06

**Authors:** Vladislav V. Nikol’skiy, Alexey M. Starosotnikov, Alexey K. Fedorenko, Ivan V. Fedyanin, Artem N. Fakhrutdinov, Maxim A. Bastrakov

**Affiliations:** 1 N.D. Zelinsky Institute of Organic Chemistry, Russian Academy of Sciences, Leninsky prosp. 47, Moscow 119991, Russia; mn181069@mail.ru (V.V.N.); alexey41@list.ru (A.M.S.); alexeyfedorenko21@mail.ru (A.K.F.); fak.part@gmail.com (A.N.F.); 2 A.N. Nesmeyanov Institute of Organoelement Compounds, Vavilova Str. 28, Moscow 119991, Russia; octy@xrlab.ineos.ac.ru

**Keywords:** nitro group, nucleophilic aromatic substitution, Smiles rearrangement, pyrido[2,3-e][1,3,4]thiadiazines, pyrimido[4,5-e][1,3,4]thiadiazines, 1,5-dihydropyrazolo[4,3-e][1,3,4]thiadiazines, dithizones, UV-Vis spectroscopy

## Abstract

A convenient method is described for the synthesis of novel 2-arylazo-1,3,4-thiadiazines fused with pyridine, pyrimidine or pyrazole ring. The synthetic strategy relies on a one-pot tandem cascade that proceeds through three consecutive steps without the isolation of intermediates: (1) nucleophilic aromatic substitution (SNAr) of the chlorine atom by the dithizone nucleophile, (2) intramolecular S-N-type Smiles rearrangement involving migration of the hetaryl fragment from nitrogen to sulfur, and (3) intramolecular nucleophilic substitution of the activated nitro group, which closes the fused thiadiazine ring. The use of *ortho*-chloronitro hetarenes is essential, as the nitro group serves both as an activating substituent for the initial SNAr step and as a leaving group in the final cyclization step. UV-Vis absorption spectra of the synthesized compounds were recorded.

## 1. Introduction

1,3,4-Thiadiazine derivatives are widely studied in medicinal and synthetic organic chemistry since they show diverse biological and pharmacological activities. For example, 1,3,4-thiadiazines were studied as antimicrobial compounds [1], antidepressants in a ligation model of acute pancreatitis [2], and immunomodulators on the course of myocardial infarction [3]. Among fused compounds, 1,2,4-triazolo[3,4-b][1,3,4]thiadiazines should be mentioned as novel analgesic/anti-inflammatory [4], anticancer [5] and anti-Alzheimer compounds [6]. In addition, thieno[3,4-e][1,2,4]thiadiazine derivatives have been described as non-nucleoside HIV-1 reverse transcriptase inhibitors [7]. Benzo and pyridoannulated 1,3,4-thiadiazines were studied as heterobicyclic herbicides [8].

**Figure 1:**
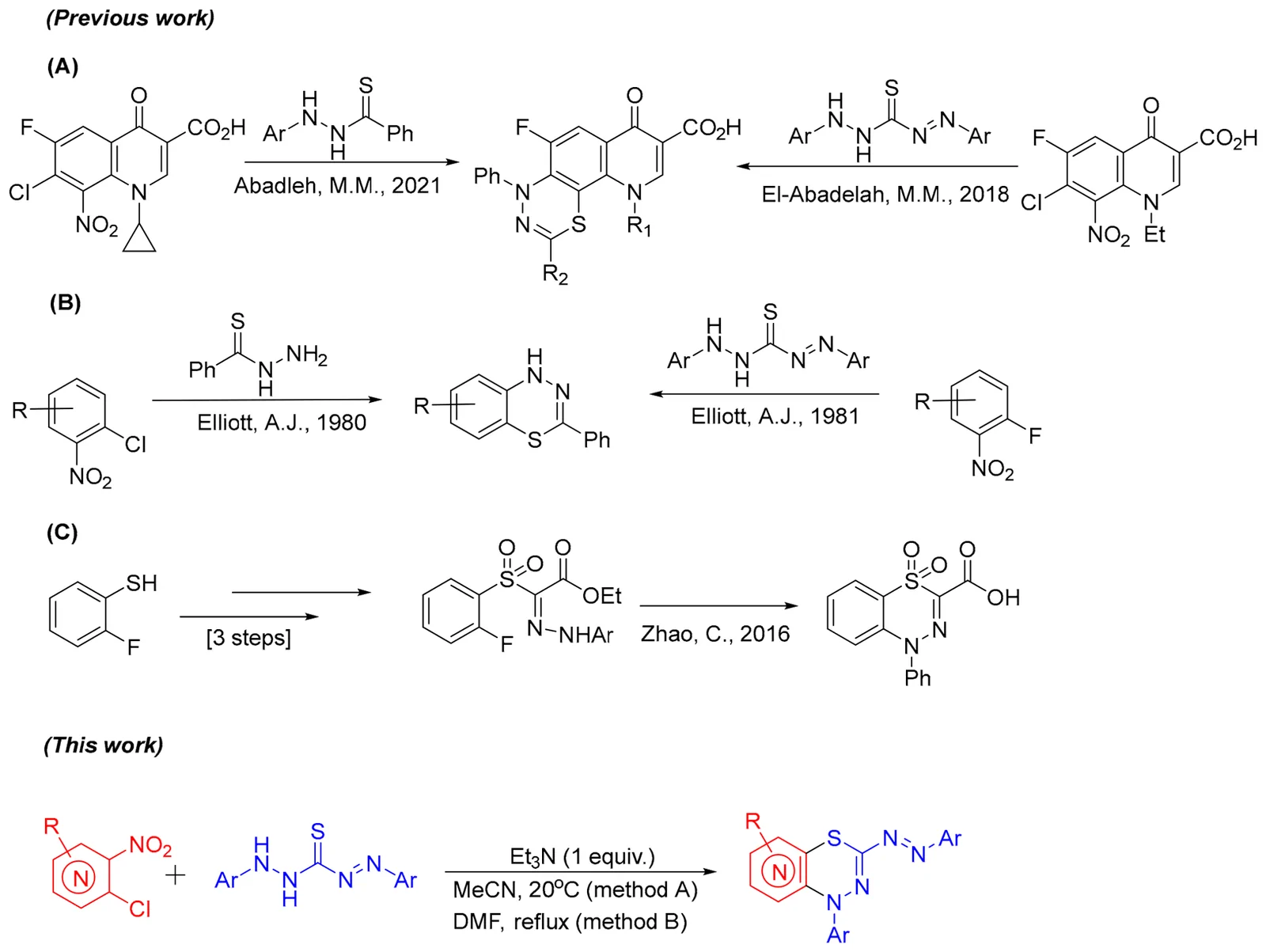
Methods for the synthesis of fused 1,3,4-thiadiazines [9,10,11,12,13] and short abstract of the present work. (**A**) Reaction of ortho-chloronitrobenzenes with N′-aryl benzohydrazides [9] and dithizones [10]. (**B**) Elliott’s synthesis of 1,3,4-benzothiadiazines from ortho-halonitrobenzenes and thiosemicarbazide/dithizones [11,12]. (**C**) Intramolecular fluorine displacement for 1,3,4-thiadiazine annulation [13].

The known methods for the synthesis of fused 1,3,4-thiadiazines are usually based on the annulation of a thiadiazine fragment to an appropriately substituted arene core, Figure 1. Thus, reactions of *ortho*-chloronitrobenzenes with N′-aryl benzohydrazides [9] or dithizones [10] resulted in the formation of 1,3,4-benzothiadiazines. A similar pathway was used by Elliott for the synthesis of several annulated 1,3,4-thiadiazines [11,12]. Intramolecular nucleophilic substitution of the fluorine atom leading to the annulation of 1,3,4-thiadiazine ring has also been described [13,14]. The use of *ortho*-chloronitro hetarenes as starting materials seems to be the most convenient and efficient method of constructing bicyclic 1,3,4-thidiazines in a reaction with S,N-binucleophiles. This approach was applied to the synthesis of novel thiazolo[4,5-b]pyridines fused with triazole or pyrimidine rings [15] and pyrido[3,2-b][1,4]benzoxazines [16]. The key steps of such methods include the nucleophilic substitution of the chlorine atom, then base-promoted Smiles rearrangement, followed by the nucleophilic aromatic substitution of the non-activated nitro group, which was recently found to be one of the best leaving groups in the pyridine series [17,18,19].

Here, we report an efficient and simple synthesis of novel 2-arylazo-1,3,4-thiadiazines fused with a pyridine, pyrimidine or pyrazole ring starting from *ortho*-chloronitro hetarenes and dithizones.

## 2. Results and Discussion

**Scheme 1:**
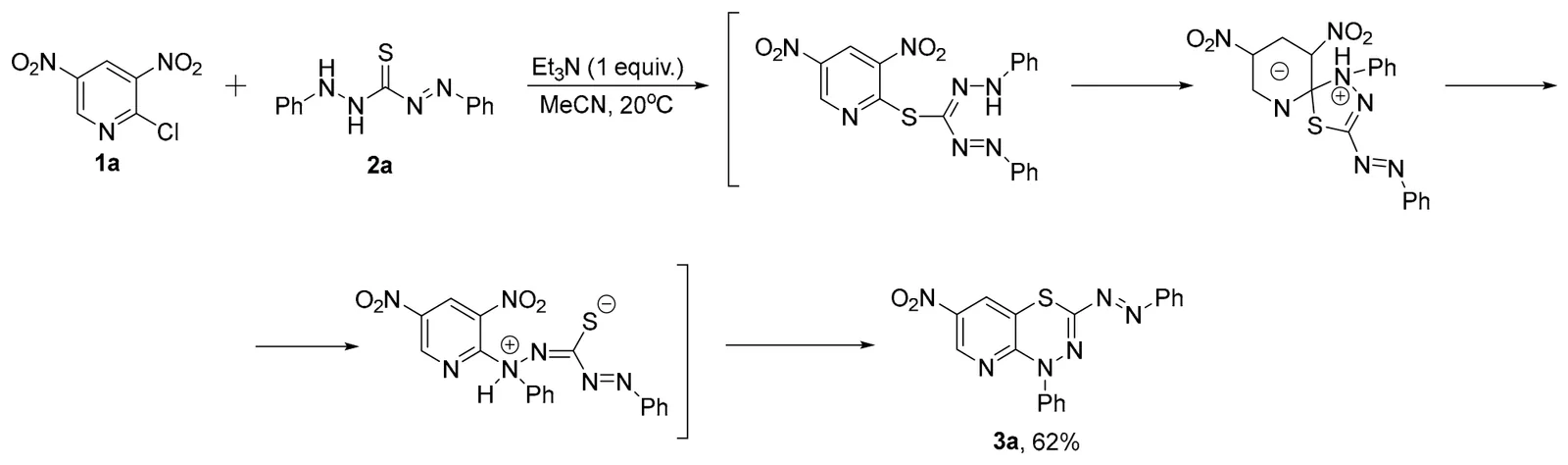
Synthesis of pyrido[2,3-e][1,3,4]thiadiazine **3a** and proposed pathway.

We found that 2-chloro-3,5-dinitropyridine **1a** readily reacts with dithizone **2a** in the presence of Et_3_N (1 equiv.) in MeCN at 20 °C to give pyrido[2,3-e][1,3,4]thiadiazine **3a** in 62% yield, Scheme 1. Apparently, the formation of **3a** may result from the base-promoted substitution of the chlorine atom with the S-nucleophilic centre of dithizone and subsequent S-N-type Smiles rearrangement followed by the nucleophilic substitution of the nitro group.

This result was not obvious since similar reactions of 2-chloro-3-nitropyridines with other S,N-binucleophiles (1,2,4-triazole-5-thiols and 4-oxopyrimidine-2-thiones) required an elevated temperature for the Smiles rearrangement to proceed [15].

**Figure 2:**
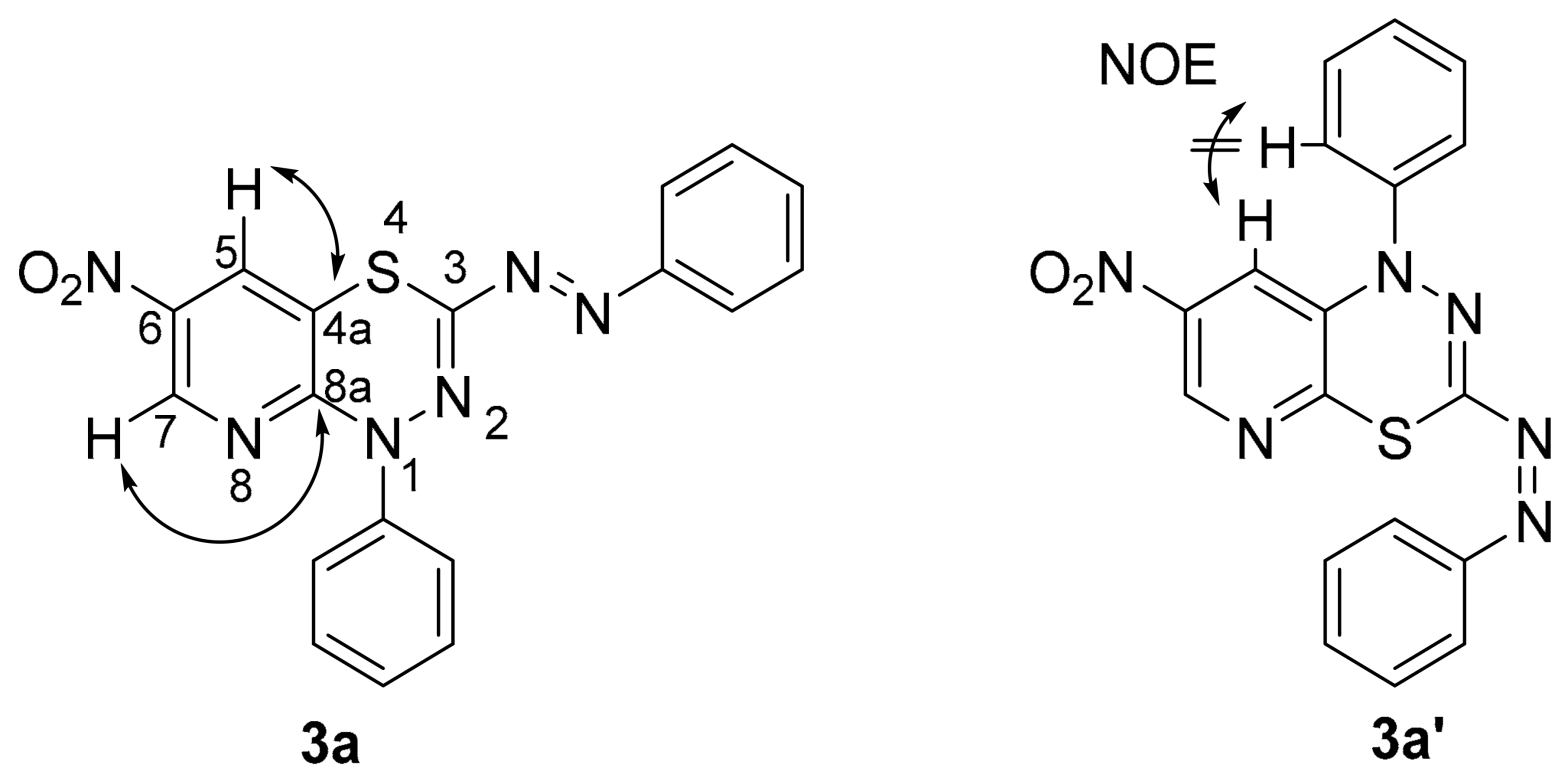
Selected correlations found in ^1^H/^13^C and NOESY spectra of **3a** and its possible isomer.

The structure elucidation of compound **3a** was made on the basis of 2D NMR experiments. 2D NOESY spectra do not contain cross-peaks corresponding to the interaction of a spatially close pyridinium proton H(5) and a phenyl ring (isomer **3a′**), Figure 1. Due to the absence of spatial contacts, this experiment proves neither the **3a** or the **3a′** isomer. Therefore, a full assignment of signals was performed using the ^1^H/^13^C HSQC and HMBC techniques. The most informative were the ^1^H/^13^C HMBC spectra, where correlations of H(7) and H(5) with C(8a) were found, Figure 2. The spin–spin coupling constants in this case are 5 Hz, which means that the carbon atom is ≤3 chemical bonds away and belongs to C(8a). In addition, low-intensity correlations of H(7) and H(5) with C(4a) were found, indicating a small spin–spin coupling constant. The quaternary carbon atoms C(4a) and C(8a) have chemical shifts of 116.55 ppm and 152.83 ppm respectively, which is in good agreement with the literature data for structurally similar pyridobenzothiazines [20].

These data allowed us to unambiguously confirm the structure of compounds **3**.

**Figure 3:**
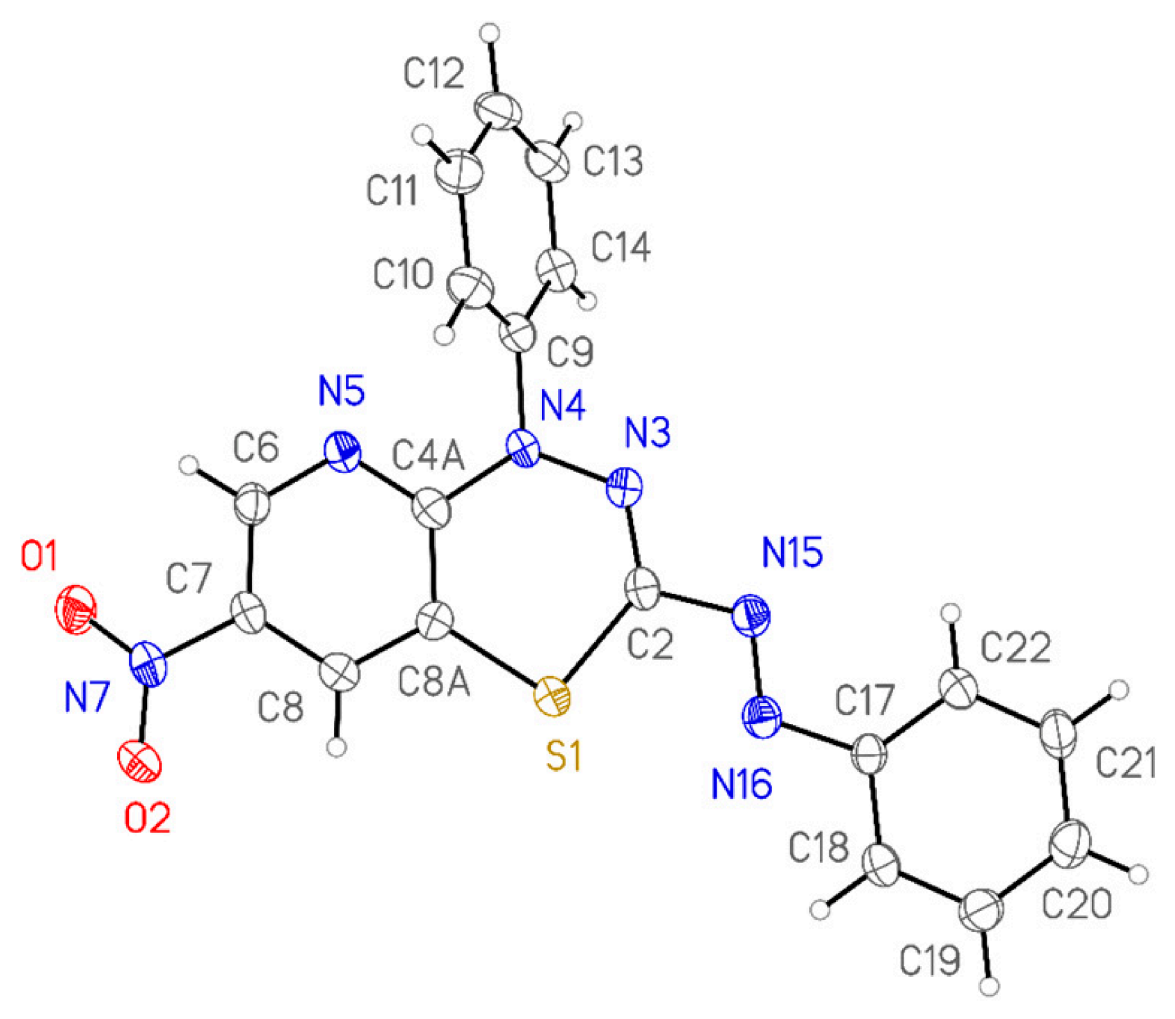
General view of **3a** in crystal. Anisotropic displacement parameters for non-hydrogen atoms are drawn at 50% probability.

Additionally X-ray analysis allowed us to unambiguously determine the structure of compound **3a**, Figure 3.

**Scheme 2:**
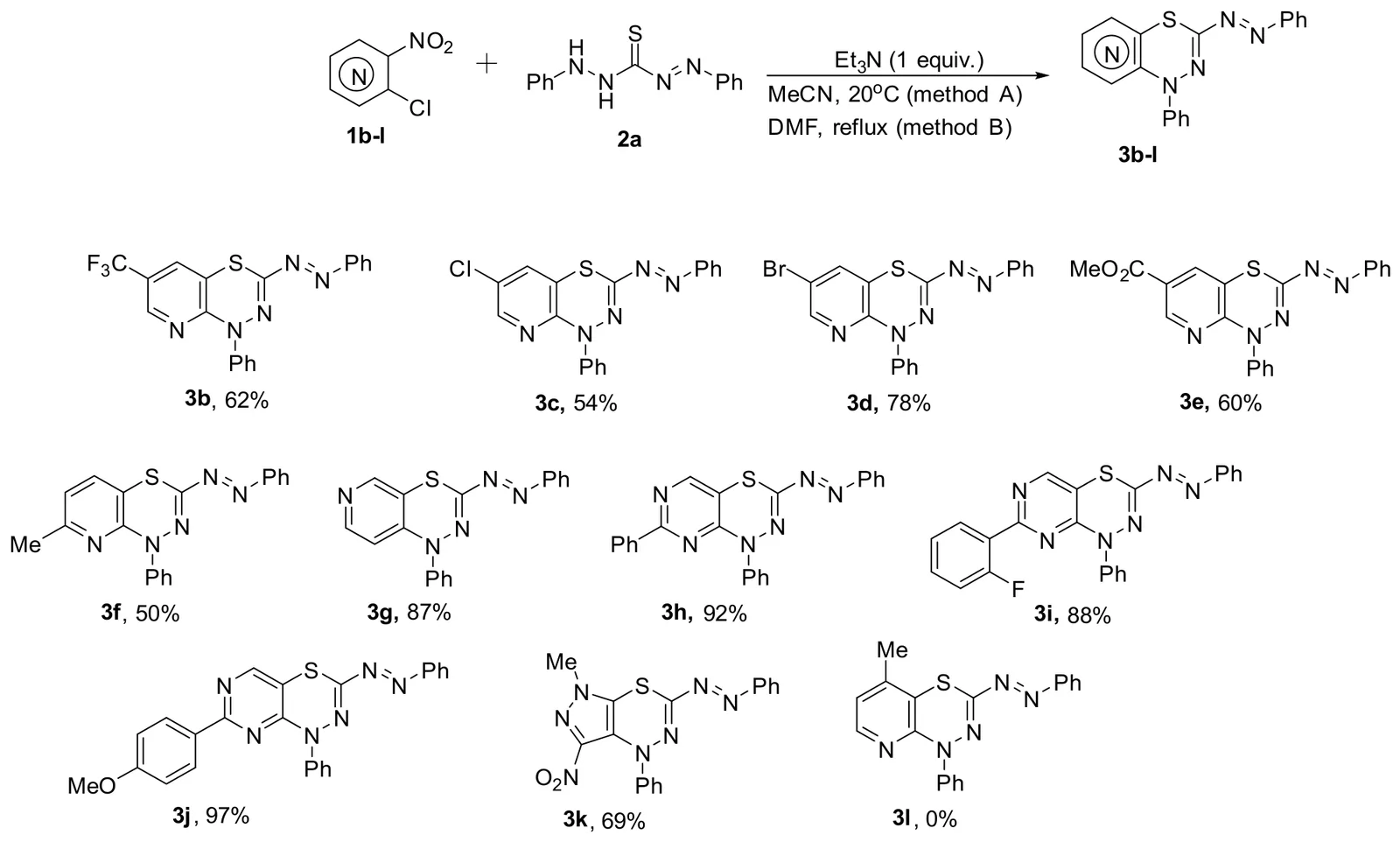
Synthesis of pyrido[2,3-e][1,3,4]thiadiazines **3b**–**k**.

To examine the scope of the reaction, a number of *ortho*-chloronitrohetarenes (**1b**–**k**) were involved in the reaction with dithizone **2a** in elaborated conditions (method A), Scheme 2. Substitution of the chlorine atom proceeded smoothly in all cases that were monitored by TLC; however, in the case of compounds **3f** and **3g**, rearrangement of the substitution product and further cyclization required much higher temperature. Therefore, these reactions were carried out under reflux in DMF. As a result, a series of 1,3,4-thiadiazines **3b**–**k** fused with a pyridine, pyrimidine and pyrazole cycle were synthesized in moderate to excellent yields, Scheme 2. An exception was observed for compound **1l**, where the target thiadiazine **3l** could not be obtained regardless of the reaction conditions employed. This outcome is presumably due to the presence of a substituent at the 4-position of the substrate. It should be noted that compound **3k** represents the first example of the novel pyrazolo[4,3-e][1,3,4]thiadiazine heterocyclic system.

**Scheme 3:**
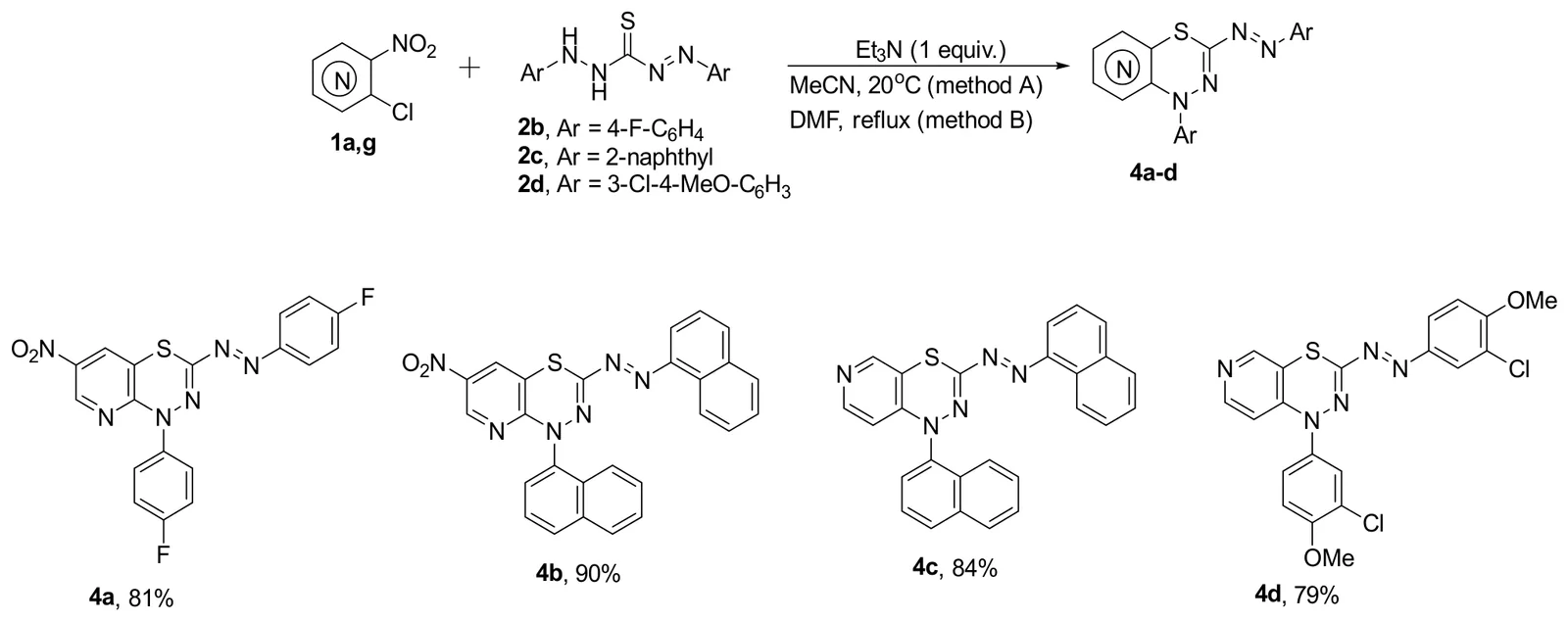
Synthesis of pyrido[2,3-e][1,3,4]thiadiazines **4a**–**d**.

Dithizone analogues **2b**–**d** were synthesized via the Bamberger route [21]. Their reactions with chloronitropyridines **1a,g** afforded fused 1,3,4-thiadiazines **4a**–**d** in high yields, Scheme 3. Compounds **4a,b** were synthesized under mild conditions by method A, whereas less electron-deficient 4-chloro-3-nitropyridine **1g** undergoes cyclization under more drastic conditions (method B). 

**Figure 4:**
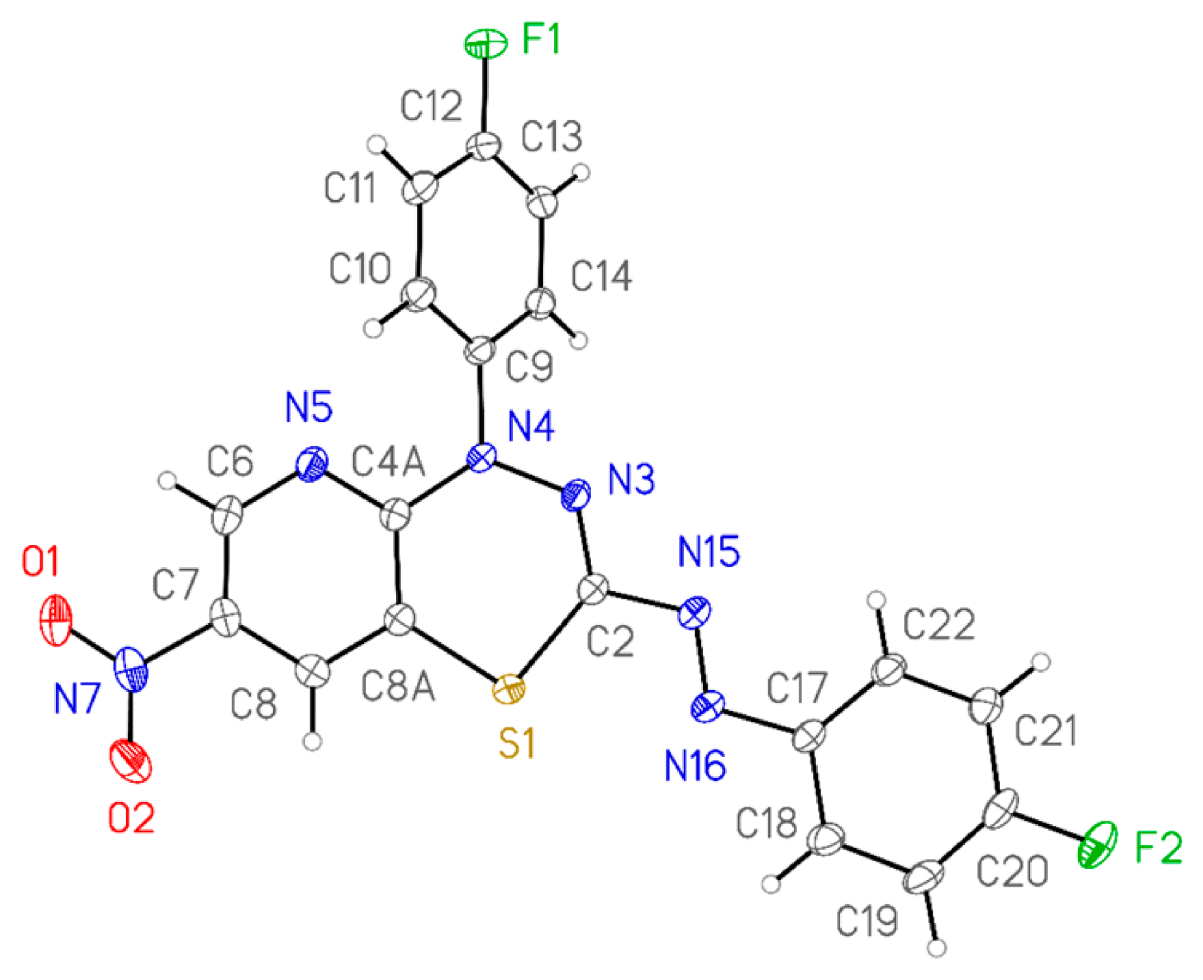
General view of **4a** in crystal. Anisotropic displacement parameters for non-hydrogen atoms are drawn at 50% probability.

The structures of compounds **4a**–**d** were confirmed by NMR spectroscopy. In the case of compound **4a**, X-ray data are available, Figure 4.

Interestingly, both compounds **3a** and **4a** contain a fused heterocyclic system of this type that, to the best of our knowledge, has not previously been characterized by single-crystal X-ray diffraction. These compounds, which differ only by two fluorine atoms, have essentially similar molecular geometries. Selected bond lengths are given in Table 1 and are consistent with the canonical formula. For each part of the fused ring system, as well as for the attached substituents, the bond lengths and angles fall within the expected ranges, as confirmed by a Mogul geometry check [22].

The relatively long C–S bonds of 1.76–1.77 Å (Table 1) suggest little or no participation of sulfur in the conjugation, whereas the remaining heterocyclic fragment forms a common conjugated system. The attached –N=N–Ph substituent also participates in this conjugated system and is approximately coplanar with it. The limited contribution of the C–S bonds to conjugation leaves this part of the heterocycle, containing formally single bonds, relatively conformationally flexible. While in **3a** the whole fused heterocyclic system is approximately planar, in **4a** the thiadiazine ring adopts a sofa conformation, with the sulfur atom displaced by 0.297(2) Å from the mean plane of the remaining fused system, corresponding to an angle of 13.80(5)° between the respective mean planes. For comparison, the corresponding values for **3a** are only 0.067(3) Å and 3.7(2)°, respectively.

The two substituents attached to atoms C4 and C7 are, as expected, more conformationally labile. The phenyl and 4-F-phenyl substituents attached to the nitrogen atom are considerably rotated relative to the heterocyclic system, with angles between the corresponding mean planes of 67.9 and 53.3°, respectively. The nitro group is also tilted relative to the heterocyclic plane, more strongly in **3a** than in **4a**, as indicated by the O1–N7–C7–C6 torsion angles of −14.2(4) and 4.75(15)°, respectively.

Despite differing by only two fluorine atoms, **3a** and **4a** exhibit completely different crystal packing. Both structures feature π-stacking contacts between their π-systems. In **3a**, the molecules form a ladder-like arrangement generated by translation along the shortest a axis. The stacking involves partial overlap of the thiadiazine and pyridine rings, as well as an interaction between the phenyl ring of the phenyldiazenyl substituent and the π-system of the N=N-containing part of a neighbouring molecule, with the shortest contact distances of C · N 3.255(4), C···C 3.382(4), and S···C 3.582(3) Å. In **4a**, in contrast, the fused heterocyclic system overlaps with its inversion-related counterpart on one side, with the shortest contact distances of C···C 3.277(2), C···N 3.290(2), and C···S 3.7582(12) Å, and with the 4-F-phenyl substituent attached to the N atom of another neighbouring molecule on the other side, primarily involving the pyridine part of the fused system (C···C contacts from 3.315(2) Å). All other intermolecular contacts are weak and non-directional in character.

Photophysical properties of substituted dithizones as well as their various metal complexes have long been attracting great scientific interest in the context of both theoretical and analytical chemistry [23,24,25]. However, to the best of our knowledge no attempts have been made so far to record UV-Vis spectra of annulated 1,3,4-thiadiazines derived from dithizones and to assess the impact of various substituents on light absorption.

**Figure 5:**
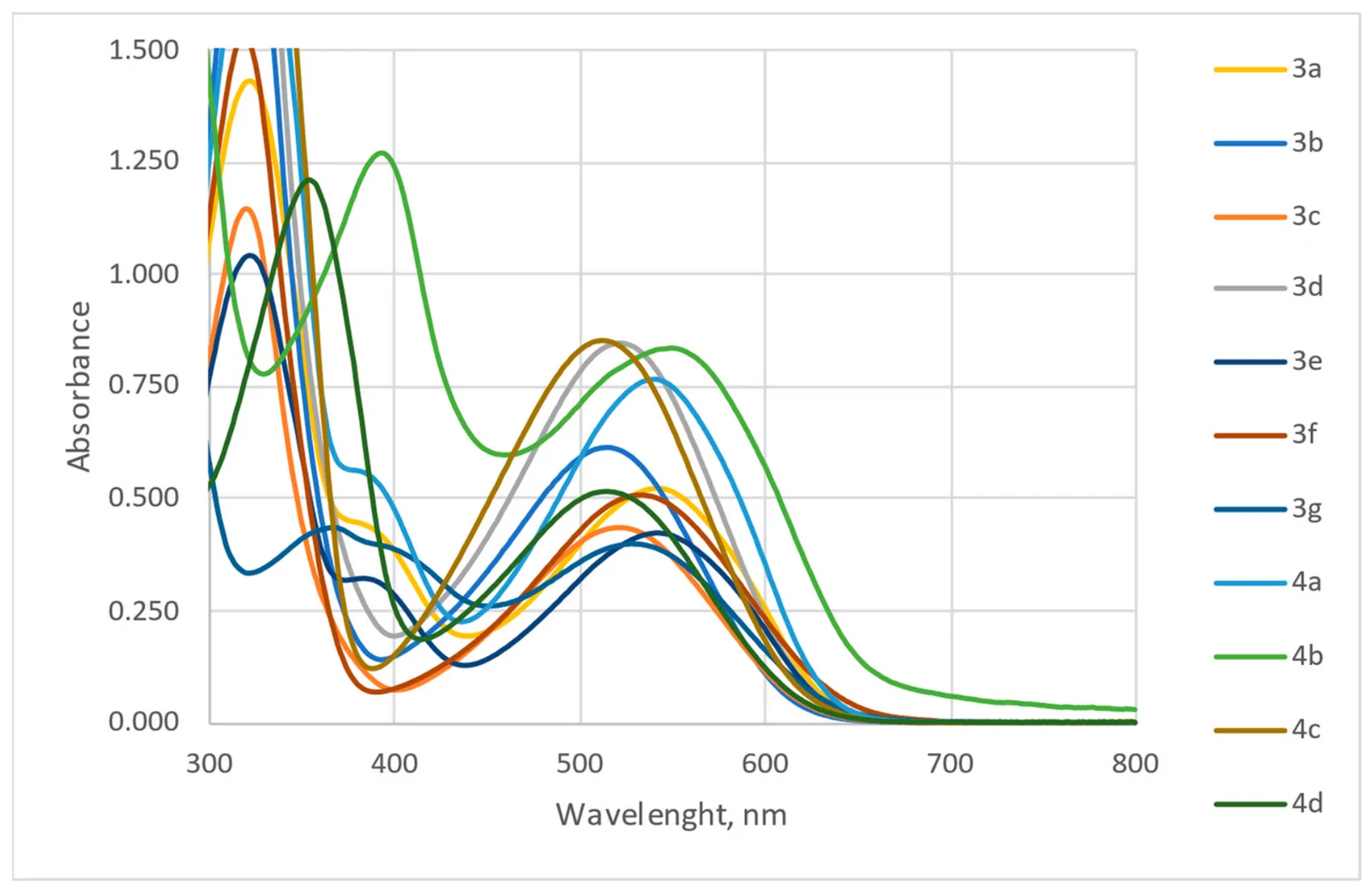
UV-Vis spectra of pyridino[1,3,4]thiadiazines **3a–g**, **4a–d** in MeCN (1 × 10^−4^ M).

**Figure 6:**
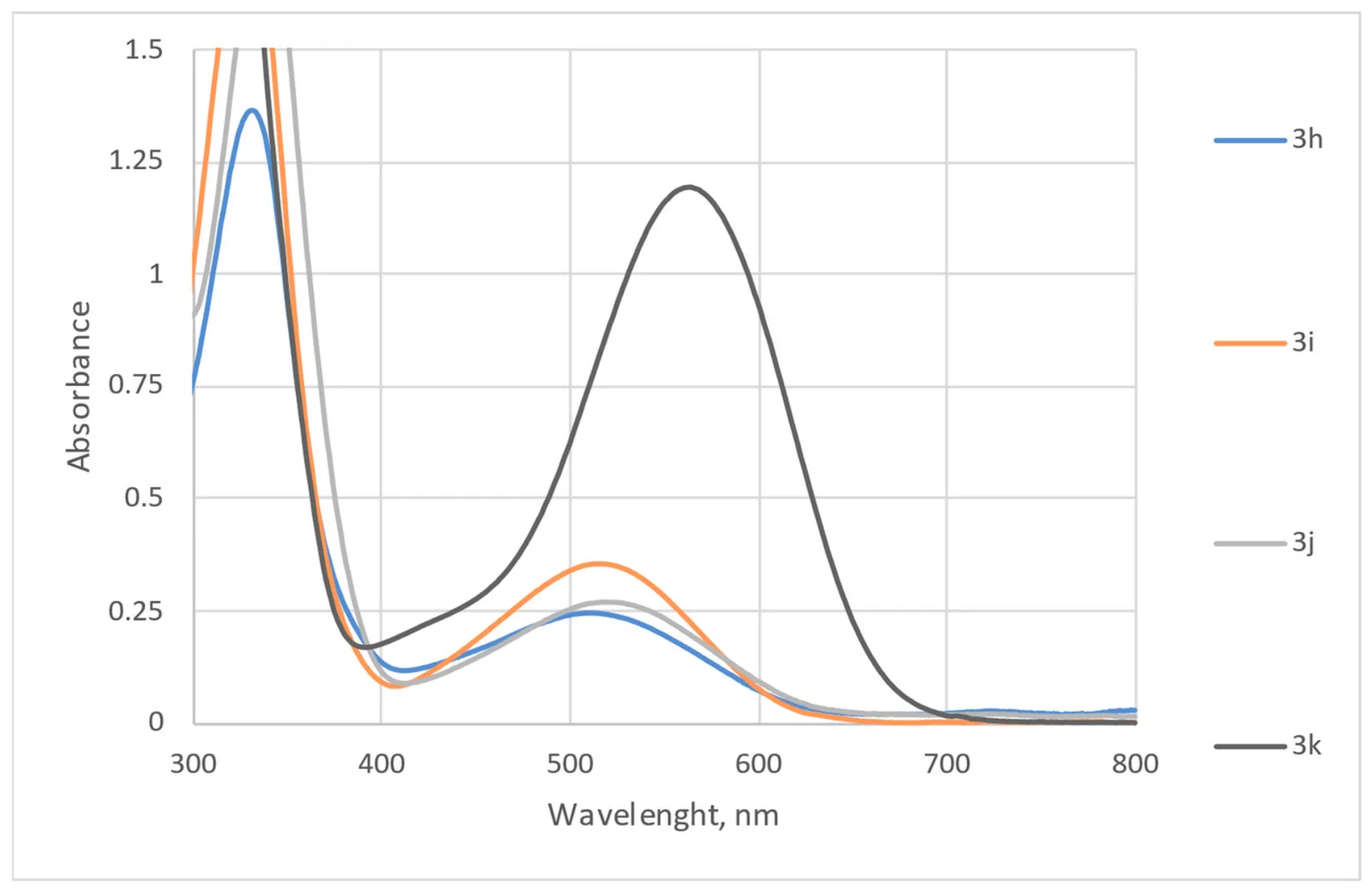
UV-Vis spectra of pyrazolo- and pyrimidino[1,3,4]thiadiazines **3h-k** in MeCN (1 × 10^−4^ M).

UV-Vis spectra of all synthesized compounds **3a–k** and **4a–d** were recorded in 10^−4^ M MeCN solution (Figure 5 and Figure 6) to assess values of λ_max_ and ε as a reference point for further research. In all cases the shortwave region (λ < 400 nm) is expectedly dominated by strong absorption of multiple aromatic rings, rendering this region of the spectrum non-informative. On the other hand, the visible region features a single strong and defined absorption peak at 510–570 nm, with the molar absorption coefficient ε ranging from 2450 to 11920 M^−1^·cm^−1^. This absorption peak is responsible for the deep purple-red colour of compounds **3a–k** and **4a–d**.

For pyridine-annulated compounds **3a**-**g** and **4a–c**, no significant correlation between substituents and absorption spectra can be pointed out except for a small yet consistent 20–30 nm red shift in λ_max_ for compounds **3a**, **3e**, **4a**, **4b**, containing a strong electron-withdrawing group in pyridine ring. 

Pyrimidine-annulated compounds **3h**, **3i** and **3j** have the lowest molar absorption coefficient values while pyrazole-annulated compound **3k** has the highest values of both λ_max_ and ε (Table 2). On the basis of these findings, we assume that the nature of the annulated cycle derived from *ortho*-chloronitroarene **1** has the largest impact on the UV-Vis absorption of 1,3,4-thiadiazines; further studies of related polycyclic systems can provide valuable insights.

## 3. Materials and Methods

### 3.1. General Information

Melting points were measured on a Stuart SMP 20 apparatus (Bibby Scientific, Staffordshire, UK). ^1^H and ^13^C NMR spectra were recorded on a Bruker AM-300 (at 300.13 and 75.13 MHz, respectively, Bruker Biospin, Ettlingen, Germany), Bruker Avance DRX 500 (at 500 and 125 MHz, respectively, Bruker Biospin, Ettlingen, Germany) or Bruker Avance II 600 spectrometer (at 600 and 150 MHz, respectively, Bruker Biospin, Ettlingen, Germany) in DMSO-d_6_ or CDCl_3_ with TMS as the internal standard. HRMS spectra were recorded on a Bruker micrOTOF II mass spectrometer (Bruker, Billerica, MA, USA) using ESI. UV-vis absorption spectra were recorded in MeCN (1 mg samples weighted with 0.0001 g accuracy and diluted to 1 × 10^−4^ M) in standard 10 × 10 × 45 mm quartz cuvettes on Cary 60 UV-Vis spectrophotometer (Agilent Technologies, Santa Clara, CA, USA). λ_max_ and ε values were calculated from single measurements with automatic baseline correction against blank solvent and reported without corrections. All reactions were monitored by TLC analysis using ALUGRAM SIL G/UV254 plates (Macherey-Nagel, Düren, Germany), which were visualized by UV light. Compounds **1a–j** and **2a** are commercially available (ABCR (Karlsruhe, Germany), Sigma Aldrich (St. Louis, MO, USA), Macklin (Shanghai, China)). Compound **1k** was synthesized according to the literature method [26].

### 3.2. General Procedure for the Synthesis of Compounds ***2b***–***d***

*Formazans:* The corresponding aniline (30 mmol) was added to a mixture of HCl, conc. (15 mL) and H_2_O (25 mL) at 0 °C. Then, NaNO_2_ (3.1 g, 45 mmol) was slowly added while stirring, reacting with the aniline to form a clear solution (30 min). The diazo solution was added to a mixture of NaOAc (60 g), AcOH (35 mL) and H_2_O (20 mL). CH_3_NO_2_ (8.6 mL, 150 mmol) was added after 10 min. The mixture was stirred for 2 h at room temperature. Then, 300 mL of H_2_O was added. The red formazyl precipitate filtered off and was washed with water and dried overnight. 

*Dithizones*: The nitroformazan (3 mmol) was suspended in 60 mL of EtOH (abs) at 35 °C. Then, (NH_4_)_2_S (20% aq, 6 mL, 17.6 mmol) was added. After 20 min (red colour fades, or precipitate forms) the mixture was poured onto crushed ice (100 mL) and a little water added to promote precipitation. The mostly dirty-white thiocarbazide was immediately filtered off and washed with water. Methanolic potassium hydroxide (2%) was intermittently poured onto the precipitate, oxidizing it to the orange-red potassium salt and then dissolving and washing it into a 250 mL flask. The solution was acidified with 2% HCl until full precipitation of the black dithizone product was effected, which was then gravity filtered through a piece of filter paper and washed with copious amounts of water. For purification purposes, the base–acid treatment was repeated.

*N,2-Bis(4-fluorophenyl)diazene-1-carbohydrazonothioic acid (***2b***)*

Yield: 55%; M.p. 163–165 °C (dec.) ^1^H NMR(Chloroform-*d*, 300 MHz): δ (ppm) 7.89–7.48 (m, 4H), 7.50–6.48 (m, 4H). 12.56 (s, 2H). HRMS: Calc. for C_13_H_10_F_2_N_4_S: 291.0510 [M + H]^+^; found: 291.0513.

*N,2-Di(naphthalen-1-yl)diazene-1-carbohydrazonothioic acid (***2c***)*

Yield: 68%; M.p. 185–187 °C; ^1^H NMR(Chloroform-*d*, 300 MHz): δ (ppm) 7.10 (d, *J* = 8.5 Hz, 2H), 7.43–8.11 (m, 8H), 8.42 (s, 2H), 8.92 (d, *J* = 8.4 Hz, 2H). HRMS: Calc. for C_21_H_15_N_4_S: 355.1012 [M + H]^+^; found: 355.1013.

*N,2-Bis(3-chloro-4-methoxyphenyl)diazene-1-carbohydrazonothioic acid (***2d***)*

Yield: 46%; M.p. 157–158 °C; ^1^H NMR(Chloroform-*d*, 300 MHz): δ (ppm) 3.98 (s, 6H), 6.66–8.28 (m, 8H), 12.42 (s, 2H) HRMS: Calc. for C_15_H_13_Cl_2_N_4_O_2_S 383.0131 [M + H]^+^; found: 383.0130.

### 3.3. General Procedure for the Synthesis of Compounds ***3a***–***k***, ***4a***–***d***

**Method A**

To a solution of compound **1** (1 mmol) and corresponding dithizone **2** (1 mmol) in MeCN (15 mL), 0.14 mL Et_3_N was added. The mixture was stirred at 20 °C (3–24 h) until complete conversion of the starting chloride (TLC monitoring), poured into water (75 mL), acidified with conc. HCl to pH 3 and the resulting precipitate was filtered off, washed with water and purified by column chromatography on MN Kieselgel 60 (Macherey-Nagel, Düren, Germany) (0.04–0.063 mm/230–400 mesh) using CHCl_3_ as an eluent to give the target compound.

**Method B**

To a solution of compound **1** (1 mmol) and corresponding dithizone **2** (1 mmol) in DMF (15 mL), 0.14 mL Et_3_N was added. The mixture was stirred under reflux for 4 h (TLC monitoring), poured into water (75 mL), acidified with conc. HCl to pH 3, and the resulting precipitate was filtered off, washed with water and purified by column chromatography on MN Kieselgel 60 (0.04–0.063 mm/230–400 mesh) using CHCl_3_ as an eluent to give the target compound.

*(E)-6-Nitro-1-phenyl-3-(phenyldiazenyl)-1H-pyrido[2,3-e][1,3,4]* *thiadiazine (***3a***):*

**Method A.** Yield: 62%; M.p. 229–230 °C; ^1^H NMR (300 MHz, CDCl_3_) δ: 7.36–7.48 (m, 1H), 7.50–7.69 (m, 7H), 7.88–8.06 (m, 3H), 8.66 (d, *J* = 2.4 Hz, 1H) ^13^C NMR (126 MHz, CDCl_3_) δ: 116.55, 123.98, 125.98, 128.14, 129.22, 129.43, 129.91, 133.44, 141.03, 141.73, 143.08, 148.98, 151.14, 152.83. HRMS: Calc. for C_18_H_13_N_6_O_2_S: 377.0806 [M + H]^+^; found: 377.0815.

*(E)-1-Phenyl-3-(phenyldiazenyl)-6-(trifluoromethyl)-1H-pyrido[2,3-e][1,3,4]* *thiadiazine (***3b***):*

**Method A.** Yield: 62%; M.p. 195–196 °C; ^1^H NMR (300 MHz, CDCl_3_) δ: 7.39–7.43 (m, 2H), 7.50–7.55 (m, 5H), 7.59–7.63 (m, 2H), 7.94–7.97 (m, 2H), 8.09 (d, 1H, *J* = 1.0 Hz). ^13^C NMR (150.9 MHz, CDCl_3_) δ: 124.0 (q, ^2^*J*_C–F_ = 33.7 Hz), 124.5, 126.6, 128.4, 129.8, 130.1, 133.1 (q, ^3^*J*_C–F_ = 3.2 Hz), 133.7, 142.8, 144.1 (q, ^3^*J*_C–F_ = 4.3 Hz), 148.8, 151.9, 152.8. HRMS: Calc. for C_19_H_13_F_3_N_5_S: 400.0843 [M + H]^+^; found: 400.0840.

*(E)-6-Chloro-1-phenyl-3-(phenyldiazenyl)-1H-pyrido[2,3-e][1,3,4]* *thiadiazine (***3c***):*

**Method A.** Yield: 54%; M.p. 217–218 °C; ^1^H NMR (300 MHz, CDCl_3_) δ: 7.22 (d, 1H, *J* = 2.2 Hz), 7.40 (t, 1H, *J* = 7.3 Hz), 7.48–7.53 (m, 5H), 7.60 (d, 2H, *J* = 7.8 Hz), 7.79 (d, 1H, *J* = 2.2 Hz), 7.92–7.96 (m, 2H). ^13^C NMR (150.9 MHz, CDCl_3_) δ: 117.3, 123.6, 125.7, 127.3, 127.5, 128.9, 129.3, 132.6, 134.9, 142.2, 143.9, 146.9, 148.0, 151.2. HRMS: Calc. for C_18_H_13_ClN_5_S: 366.0580 [M + H]^+^; found: 366.0577.

*(E)-6-Bromo-1-phenyl-3-(phenyldiazenyl)-1H-pyrido[2,3-e][1,3,4]thiadiazine (***3d***)*:

**Method A.** Yield: 78%; M.p. 206–207 °C; ^1^H NMR (300 MHz, CDCl_3_) δ: 7.34–7.39 (m, 2H), 7.48–7.61 (m, 7H), 7.88–7.95 (m, 3H). ^13^C NMR (126 MHz, CDCl_3_) δ: 115.1, 117.6, 123.7, 125.8, 127.3, 128.9, 129.3, 132.6, 137.4, 142.1, 146.2, 147.1, 148.4, 151.2. HRMS: Calc. for C_18_H_13_BrN_5_S: 410.0075 [M + H]^+^; found: 410.0070.

*Methyl (E)-1-phenyl-3-(phenyldiazenyl)-1H-pyrido[2,3-e][1,3,4]* *thiadiazine-6-carboxylate (* ***3e*** *):*

**Method A.** Yield: 60%; M.p. 235–237 °C; ^1^H NMR (300 MHz, CDCl_3_) δ: 3.90 (s, 3H), 7.39 (t, *J* = 7.3 Hz, 1H), 7.47–7.58 (m, 5H), 7.62 (d, *J* = 7.7 Hz, 2H), 7.78 (d, *J* = 2.0 Hz, 1H), 7.96 (dd, *J* = 6.6, 3.1 Hz, 2H), 8.46 (d, *J* = 2.0 Hz, 1H). ^13^C NMR (126 MHz, CDCl_3_) δ: 52.28, 115.15, 122.74, 123.75, 125.87, 127.56, 128.99, 129.26, 132.83, 136.09, 142.09, 148.25, 148.63, 151.21, 152.17, 164.56. HRMS: Calc. for C_20_H_16_N_5_O_2_S: 390.1019 [M + H]^+^; found: 390.1021.

*(E)-7-Methyl-1-phenyl-3-(phenyldiazenyl)-1H-pyrido[2,3-e][1,3,4]* *thiadiazine (* ***3f*** *):*

**Method B.** Yield: 50%; M.p. 202–204 °C; ^1^H NMR (300 MHz, CDCl_3_) δ: 2.25 (s, 3H), 6.71 (d, *J* = 7.6 Hz, 1H), 7.14 (d, *J* = 7.6 Hz, 1H), 7.31–7.37 (m, 1H), 7.43–7.56 (m, 5H), 7.64–7.71 (m, 2H), 7.87–8.01 (m, 2H). ^13^C NMR (126 MHz, CDCl_3_) δ: 23.60, 111.28, 120.07, 123.52, 125.46, 126.40, 128.42, 129.16, 132.15, 135.97, 142.53, 147.43, 148.62, 151.42, 155.16. HRMS: Calc. for C_19_H_16_N_5_S: 346.1121 [M + H]^+^; found: 346.1122.

*(E)-1-Phenyl-3-(phenyldiazenyl)-1H-pyrido[4,3-e][1,3,4]thiadiazine (****3g****)*:

**Method B.** Yield: 87%; M.p. 170–171 °C; ^1^H NMR(300 MHz, CDCl_3_): δ (ppm) 6.31 (d, *J* = 5.6 Hz, 1H), 7.22–8.41 (m, 12H) ^13^C NMR (75 MHz, CDCl_3_) δ 108.28, 115.45, 123.77, 125.80, 128.20, 129.29, 129.99, 132.76, 141.88, 145.61, 148.23, 149.91, 150.39, 151.35. HRMS: Calc. for C_18_H_14_N_5_S: 332.0964 [M + H]^+^; found: 332.0969.

*(E)-1,7-Diphenyl-3-(phenyldiazenyl)-1H-pyrimido[4,5-e][1,3,4]thiadiazine (****3h****)*:

**Method A.** Yield: 92%; M.p. 265–266 °C; ^1^H NMR(300 MHz, CDCl_3_): δ (ppm) 7.32–7.51 (m, 4H), 7.49–7.65 (m, 5H), 7.71 (d, *J* = 7.7 Hz, 2H), 7.89–8.04 (m, 2H), 8.09 (d, *J* = 7.6 Hz, 2H), 8.14 (s, 1H) ^13^C NMR (151 MHz, CDCl_3_) δ 111.10, 124.59, 126.44, 128.21, 128.56, 129.15, 129.47, 130.08, 130.45, 131.61, 133.80, 137.08, 142.01, 150.10, 151.90, 154.41, 155.42, 163.56. HRMS: Calc. for C_23_H_17_N_6_S: 409.1231 [M + H]^+^; found: 409.1230.

*(E)-7-(2-Fluorophenyl)-1-phenyl-3-(phenyldiazenyl)-1H-pyrimido[4,5-e][1,3,4]* *thiadiazine (* ***3i*** *):*

**Method A.** Yield: 88%; M.p. 240–241 °C; ^1^H NMR(300 MHz, CDCl_3_): δ (ppm) 6.81–8.39 (m, 15H) ^13^C NMR (126 MHz, CDCl_3_) δ 110.88, 116.84 (d, *J* = 22.8 Hz), 123.89 (d, *J* = 6.2 Hz), 125.58, 127.43, 128.66, 129.34, 131.33, 131.33, 131.90, 131.97, 133.12, 150.23 (d, *J* = 214.9 Hz), 153.54, 154.53. HRMS: Calc. for C_23_H_16_FN_6_S: 427.1136 [M + H]^+^; found: 427.1141.

*(E)-7-(4-Methoxyphenyl)-1-phenyl-3-(phenyldiazenyl)-1H-pyrimido[4,5-e][1,3,4]* *thiadiazine (* ***3j*** *):*

**Method A.** Yield: 97%; M.p. 260–261 °C; ^1^H NMR(300 MHz, CDCl_3_): δ (ppm) 3.84 (s, 3H), 6.81–6.93 (m, 2H), 7.36–7.48 (m, 1H), 7.47–7.60 (m, 6H), 7.66–7.74 (m, 2H), 7.92–8.00 (m, 2H), 8.00–8.07 (m, 2H), 8.08 (s, 1H) ^13^C NMR (126 MHz, CDCl_3_) δ 55.30, 109.08, 113.73, 123.81, 125.68, 127.36, 128.66, 129.00, 129.31, 129.51, 132.98, 141.30, 149.39, 151.12, 153.61, 154.50, 161.93, 162.57. HRMS: Calc. for C_24_H_19_N_6_OS: 439.1336 [M + H]^+^; found: 439.1341.

*(E)-5-Methyl-7-nitro-1-phenyl-3-(phenyldiazenyl)-1,5-dihydropyrazolo[4,3-e][1,3,4]* *thiadiazine (***3k***):*

**Method A.** Yield: 69%; M.p. 235–236 °C; ^1^H NMR (300 MHz, CDCl_3_) δ: 3.85 (s, 3H), 7.44 (br.s, 5H), 7.55 (br.s, 3H), 7.94 (br.s, 2H). ^13^C NMR (126 MHz, CDCl_3_) δ 37.56, 111.56, 113.29, 120.54, 123.73, 126.57, 127.96, 129.28, 129.36, 132.73, 143.57, 143.75, 150.82. HRMS: Calc. for C_17_H_14_N_7_O_2_S: 380.0930 [M + H]^+^; found: 380.0920.

*(E)-1-(4-Fluorophenyl)-3-((4-fluorophenyl)diazenyl)-6-nitro-1H-pyrido[2,3-e][1,3,4]* *thiadiazine (* ***4a*** *):*

**Method A.** Yield: 81%; M.p. 239–240 °C; ^1^H NMR(300 MHz, CDCl_3_): δ (ppm) 7.14–7.30 (m, 4H), 7.49–7.62 (m, 2H), 7.90–8.05 (m, 3H), 8.65 (d, *J* = 2.4 Hz, 1H). ^13^C NMR (75 MHz, CDCl_3_) δ 116.20 (d, *J* = 23.2 Hz), 116.70 (d, *J* = 23.1 Hz), 126.31 (d, *J* = 9.4 Hz), 127.96 (d, *J* = 8.7 Hz), 129.98, 137.72, 141.10, 143.03, 147.74, 148.89, 152.73, 161.84 (d, *J* = 248.2 Hz), 165.88 (d, *J* = 257.2 Hz). HRMS: Calc. for C_18_H_11_F_2_N_6_O_2_S: 413.0616 [M + H]^+^; found: 413.0627. 

*(E)-1-(Naphthalen-1-yl)-3-(naphthalen-1-yldiazenyl)-6-nitro-1H-pyrido[2,3-e][1,3,4]thiadiazine (****4b****)*:

**Method A.** Yield: 90%; M.p. 240 (dec.); ^1^H NMR(500 MHz, CDCl_3_): δ (ppm) 7.60 (d, *J* = 9.1 Hz, 2H), 7.67 (s, 1H), 7.77 (d, *J* = 7.3 Hz, 2H), 7.91–8.15 (m, 9H), 8.53 (d, *J* = 2.4 Hz, 1H), 8.86 (d, *J* = 8.4 Hz, 1H) HRMS: Calc. for C_26_H_17_N_6_O_2_S: 477.1127 [M + H]^+^; found: 477.1128.

*(E)-1-(Naphthalen-1-yl)-3-(naphthalen-1-yldiazenyl)-1H-pyrido[4,3-e][1,3,4]* *thiadiazine (* ***4c*** *):*

**Method B.** Yield: 84%; M.p. 202–203 °C; ^1^H NMR(300 MHz, DMSO-*d*_6_): δ (ppm) 5.83 (d, *J* = 5.7 Hz, 1H), 7.56–7.86 (m, 7H), 7.88–8.02 (m, 2H), 8.08 (d, *J* = 8.0 Hz, 1H), 8.11–8.26 (m, 4H), 8.31 (s, 1H), 8.76 (d, *J* = 8.4 Hz, 1H) ^13^C NMR (75 MHz, DMSO-*d*_6_) δ 108.63, 113.31, 115.17, 122.67, 122.77, 126.46, 126.65, 126.96, 127.56, 127.63, 128.38, 128.63, 128.88, 129.03, 129.36, 130.48, 131.07, 134.48, 134.98, 137.64, 145.24, 145.87, 146.33, 149.60, 150.41. HRMS: Calc. for C_26_H_18_N_5_S: 432.1277 [M + H]^+^; found: 432.1284.

*(E)-1-(3-Chloro-4-methoxyphenyl)-3-((3-chloro-4-methoxyphenyl)diazenyl)-1H-pyrido[4,3-e][1,3,4]* *thiadiazine (* ***4d*** *):*

**Method B.** Yield: 79%; M.p. 223–224 °C; ^1^H NMR(300 MHz, CDCl_3_): δ (ppm) 4.00 (d, *J* = 6.8 Hz, 6H), 6.25 (d, *J* = 5.5 Hz, 1H), 7.06 (dd, *J* = 8.8, 4.8 Hz, 2H), 7.44 (dd, *J* = 8.8, 2.7 Hz, 1H), 7.62 (d, *J* = 2.6 Hz, 1H), 7.91 (dd, *J* = 8.8, 2.4 Hz, 2H), 8.00–8.20 (m, 3H). ^13^C NMR (75 MHz, CDCl_3_) δ 29.70, 56.52, 56.60, 108.07, 111.65, 112.58, 115.10, 123.67, 124.03, 125.58, 126.21, 128.28, 135.13, 145.51, 145.68, 148.11, 149.97, 150.45, 154.95, 158.68. HRMS: Calc. for C_20_H_15_Cl_2_N_5_O_2_S: 460.0396 [M + H]^+^; found: 460.0399.

### 3.4. X-Ray Crystallographic Data and Refinement Details

Data collection was performed on a Bruker APEX II diffractometer (Bruker, Billerica, MA, USA) equipped with a Photon 2 detector and operating with MoKα radiation (λ = 0.71073 Å). Frames were integrated using the Bruker SAINT software package (Version 6.45) [27] by a narrow-frame algorithm. A semi-empirical absorption correction was applied with the SADABS program [28] using the intensity data of equivalent reflections. The structures were solved with a dual-space approach with SHELXT program [29] and refined by the full-matrix least-squares technique against F^2^_hkl_ in anisotropic approximation with the SHELXL-2019/3 [30] software package. All hydrogen atoms were placed in calculated positions and refined in the riding model with U_iso_(H) constrained to be 1.2U_eq_ of the connected carbon atoms. Detailed crystallographic information is given in Table S1 (see in Supplementary Information). Crystallographic data have been deposited to the Cambridge Crystallographic Data Centre, CCDC 2580925-2580926, and can be retrieved free of charge via https://www.ccdc.cam.ac.uk/structures, accessed on 30 September 2026.

## 4. Conclusions

Thus, we have developed a method for the synthesis a series of new 1H-pyrido[2,3-e][1,3,4]thiadiazines and 1H-pyrimido[4,5-e][1,3,4]thiadiazines **3a–j** and **4a–d** as well as representative of new heterocyclic system: 5-Methyl-7-nitro-1-phenyl-3-(phenyldiazenyl)-1,5-dihydropyrazolo[4,3-*e*][1,3,4]thiadiazine **3k.** The method is based on the interaction of *ortho*-chloro nitro heteroarenes with different dithizones, acting as binucleophiles proceeding through the nucleophilic substitution of the chlorine atom and subsequent Smiles rearrangement, followed by the nucleophilic substitution of the nitro group. UV-Vis absorption spectra of the synthesized compounds were recorded.

## Figures and Tables

**Table 1:** Selected bond lengths in compounds **3a** and **4a.**

Bond	3a	4a
S1-C8A	1.761(3)	1.7636(10)
S1-C2	1.771(3)	1.7602(10)
C2-N3	1.293(4)	1.2849(13)
N3-N4	1.361(3)	1.3672(11)
N4-C4A	1.390(4)	1.3977(13)
N4-C9	1.450(4)	1.4378(12)
C4A-N5	1.338(4)	1.3318(12)
C4A-C8A	1.413(4)	1.4089(13)
N5-C6	1.333(4)	1.3403(13)
C6-C7	1.374(4)	1.3731(15)
N7-C7	1.449(4)	1.4565(13)
C7-C8	1.381(4)	1.3918(14)
C8-C8A	1.382(4)	1.3771(14)
C2-N15	1.403(4)	1.4024(12)
N15-N16	1.276(3)	1.2708(12)

**Table 2:** Absorbance maximums of fused 1,3,4-thiadiazines in the visible region.

Compound	λ_max_ nm	ε_1_ M^−1^·cm^−1^
**3a**	543	5230
**3b**	514	6160
**3c**	522	4360
**3d**	523	4900
**3e**	543	4220
**3f**	532	5080
**3g**	528	4020
**3h**	510	2450
**3i**	516	3560
**3j**	515	2670
**3k**	565	11920
**4a**	542	7680
**4b**	550	8340
**4c**	512	8550
**4d**	514	5170

## Data Availability

The original contributions presented in this study are included in the article/Supplementary Materials. Further inquiries can be directed to the corresponding author(s).

## Supplementary Materials

The following supporting information can be downloaded at: https://www.mdpi.com/article/10.3390/ijms27198886/s1.
