# Supplementary material for: Simple Synthesis of Fused 1,3,4-Thiadiazines Through Successive SNAr Processes
Source: Int J Mol Sci. 2026 Oct 6;27(19):8886. doi: 10.3390/ijms27198886 (PMC13665132; doi:10.3390/ijms27198886)

checkCIF/PLATON report

Structure factors have been supplied for datablock(s) bm1615

THIS REPORT IS FOR GUIDANCE ONLY. IF USED AS PART OF A REVIEW PROCEDURE FOR PUBLICATION, IT SHOULD NOT REPLACE THE EXPERTISE OF AN EXPERIENCED CRYSTALLOGRAPHIC REFEREE.

No syntax errors found. CIF dictionary Interpreting this report

Datablock: bm1615

Bond precision: C-C = 0.0046 A Wavelength=0.71073

Cell:	a=4.5363(13)	b=18.554(5)	c=19.955(6)
	alpha=90	beta=91.429(7)	gamma=90
Temperature:	120 K		

	Calculated	Reported
Volume	1679.0(8)	1679.1(8)
Space group	P 21/n	P 21/n
Hall group	-P 2yn	-P 2yn
Moiety formula	C18 H12 N6 O2 S	C18 H12 N6 O2 S
Sum formula	C18 H12 N6 O2 S	C18 H12 N6 O2 S
Mr	376.40	376.40
Dx,g cm-3	1.489	1.489
Z	4	4
Mu (mm-1)	0.221	0.221
F000	776.0	776.0
F000'	776.77	
h,k,lmax	5,22,24	5,22,24
Nref	3313	3311
Tmin,Tmax	0.971,0.989	0.781,0.862
Tmin'	0.932	

Correction method= # Reported T Limits: Tmin=0.781 Tmax=0.862
AbsCorr = MULTI-SCAN

Data completeness= 0.999 Theta(max)= 25.992

R(reflections)= 0.0610(2063) wR2(reflections)= 0.1529(3311)

S = 0.994 Npar= 245

The following ALERTS were generated. Each ALERT has the format
test-name_ALERT_alert-type_alert-level.
Click on the hyperlinks for more details of the test.

Alert level C

```
PLAT340_ALERT_3_C Low Bond Precision on  C-C Bonds ..............    0.00463 Ang.
PLAT430_ALERT_2_C Short Inter D...A Contact  O2       ..O2       .       2.88 Ang.
                                              -x,1-y,1-z  =      3_566 Check
PLAT906_ALERT_3_C Large K Value in the Analysis of Variance ......      7.173 Check
```

Alert level G

```
PLAT910_ALERT_3_G Missing FCF Reflection(s) Below Theta(Min)[Deg]=       2.04 Note
                0  1  1,
PLAT912_ALERT_4_G Missing # of FCF Reflections Above STh/L=  0.600          1 Note
PLAT933_ALERT_2_G Number of HKL-OMIT Records in Embedded .res File          1 Note
                0  1  1,
PLAT969_ALERT_5_G The 'Henn et al.' R-Factor-gap value ..........      1.550 Note
              Predicted wR2: Based on SigI**2  9.87 or SHELX Weight 15.39
PLAT978_ALERT_2_G Number C-C Bonds with Positive Residual Density.          2 Info
PLAT994_ALERT_1_G SHELXL .ins Contains no or MERG 0 Instruction ..          ! Note
```

---

0 **ALERT level A** = Most likely a serious problem - resolve or explain
0 **ALERT level B** = A potentially serious problem, consider carefully
3 **ALERT level C** = Check. Ensure it is not caused by an omission or oversight
6 **ALERT level G** = General information/check it is not something unexpected

1 ALERT type 1 CIF construction/syntax error, inconsistent or missing data
3 ALERT type 2 Indicator that the structure model may be wrong or deficient
3 ALERT type 3 Indicator that the structure quality may be low
1 ALERT type 4 Improvement, methodology, query or suggestion
1 ALERT type 5 Informative message, check

It is advisable to attempt to resolve as many as possible of the alerts in all categories. Often the minor alerts point to easily fixed oversights, errors and omissions in your CIF or refinement strategy, so attention to these fine details can be worthwhile. It is up to the individual to critically assess their own results and, if necessary, seek expert advice.

PLATON version of 15/01/2026; check.def file version of 02/01/2026

duplicate check

No duplication found

Datablock bm1615 - ellipsoid plot

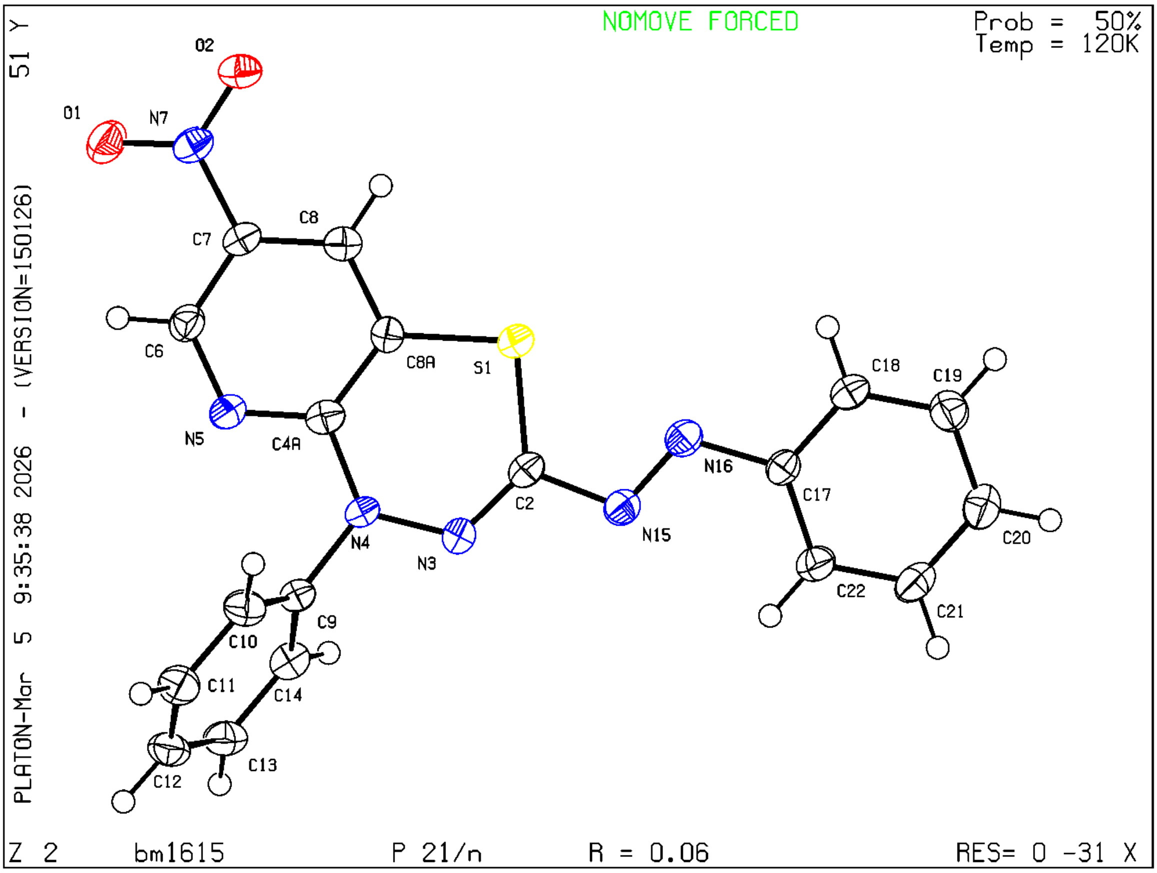

Supplement: Supplementary file 1 [file ijms-27-08886-s001.zip › bm1615.checkcif.pdf]
