# Supplementary material for: Simple Synthesis of Fused 1,3,4-Thiadiazines Through Successive SNAr Processes
Source: Int J Mol Sci. 2026 Oct 6;27(19):8886. doi: 10.3390/ijms27198886 (PMC13665132; doi:10.3390/ijms27198886)

Table of contents

NMR spectra for **3a**	S2
NMR spectra for **3b**	S3
NMR spectra for **3c**	S4
NMR spectra for **3d**	S5
NMR spectra for **3e**	S6
NMR spectra for **3f**	S7
NMR spectra for **3g**	S8
NMR spectra for **3h**	S9
NMR spectra for **3i**	S10
NMR spectra for **3j**	S11
NMR spectra for **3k**	S12
NMR spectra for **4a**	S13
NMR spectra for **4b**	S14
NMR spectra for **4c**	S15
NMR spectra for **4d**	S16
Crystallographic data for **3a** and **4a**.	S17
X-Ray data for **3a**	S18-S20
X-Ray data for **4a**	S21-S23

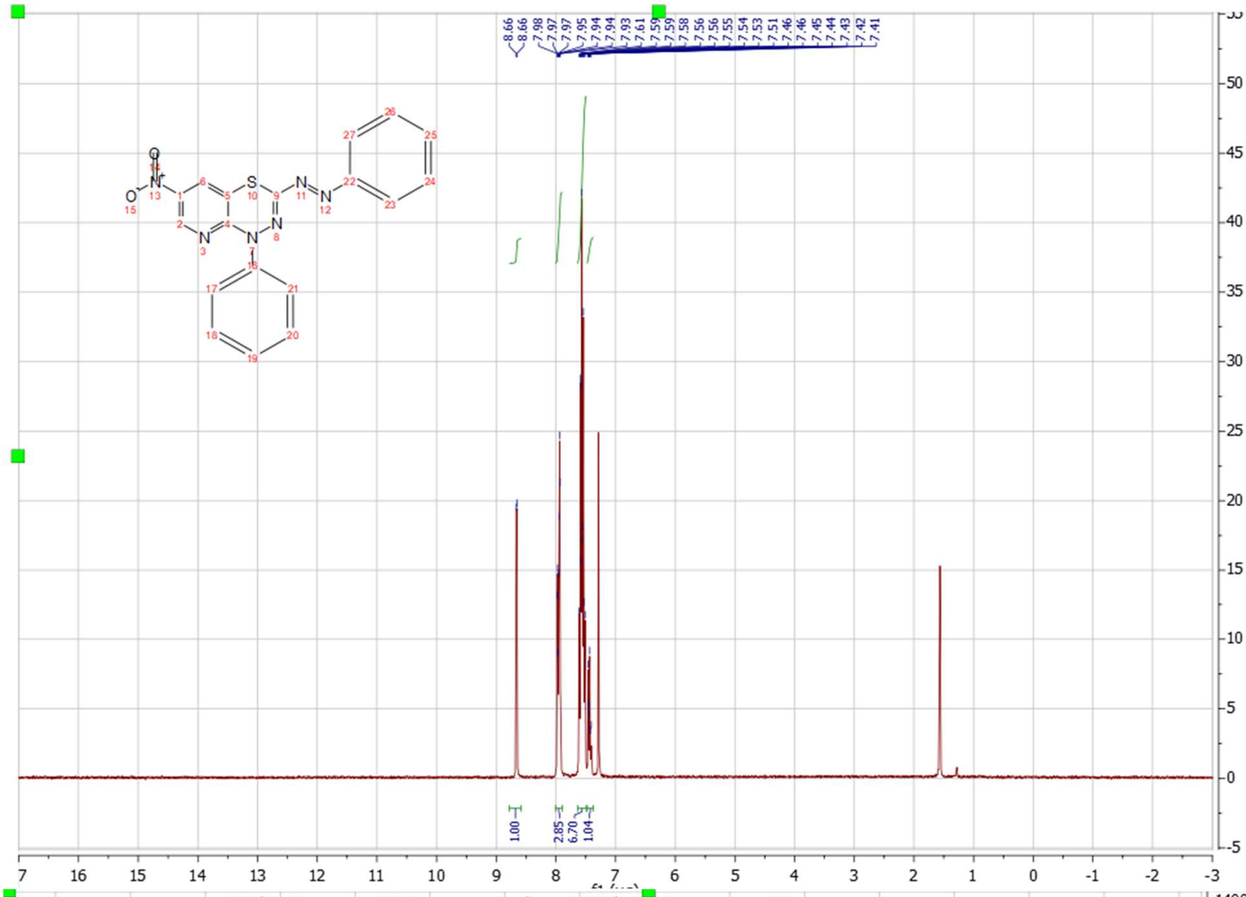
8.66
8.66
7.98
7.97
7.97
7.95
7.94
7.94
7.93
7.61
7.59
7.59
7.58
7.56
7.56
7.55
7.54
7.53
7.51
7.46
7.45
7.44
7.43
7.42
7.41
1.00
2.85
6.70
1.04
S
N
O
15
13
10
11
12

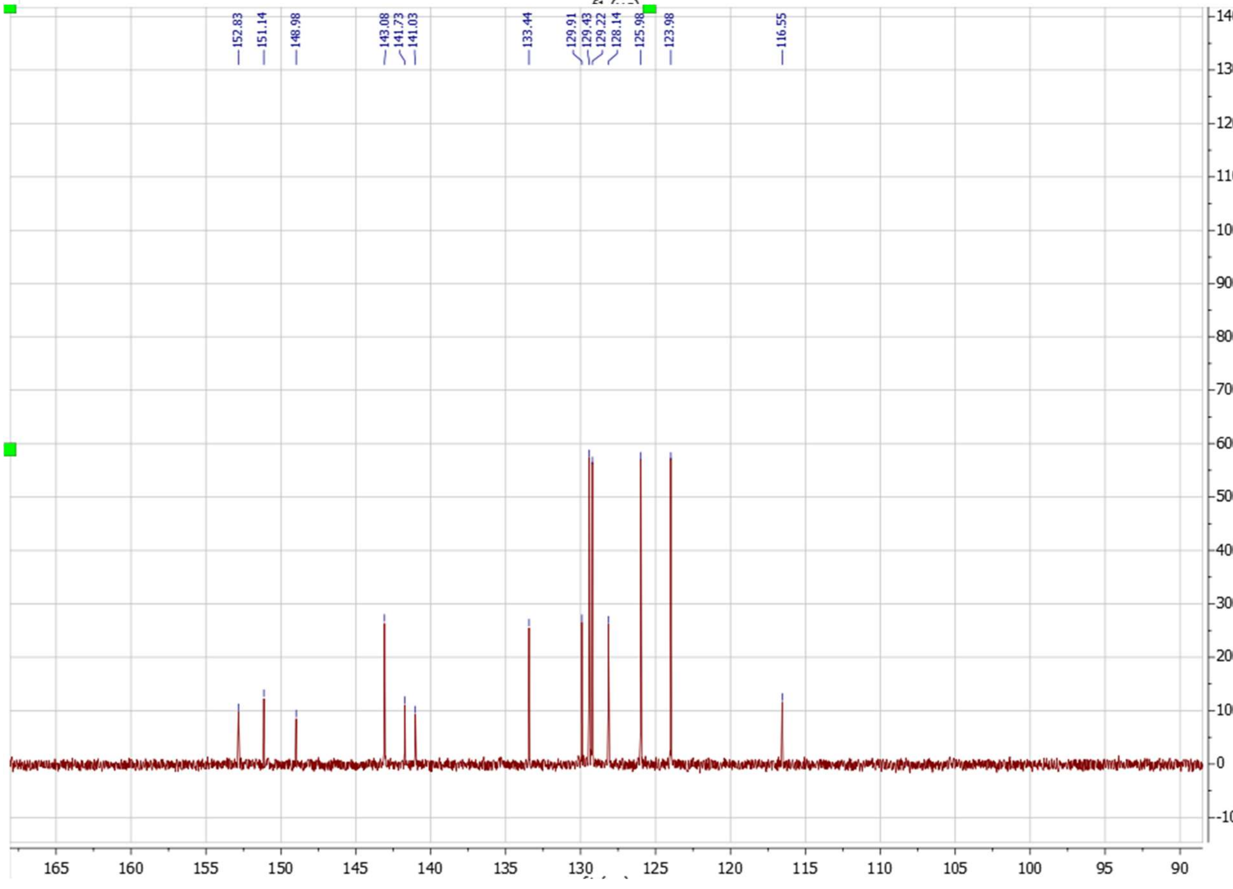
152.83
151.14
148.98
143.08
141.73
141.03
133.44
129.91
129.43
129.22
128.14
125.98
123.98
116.55

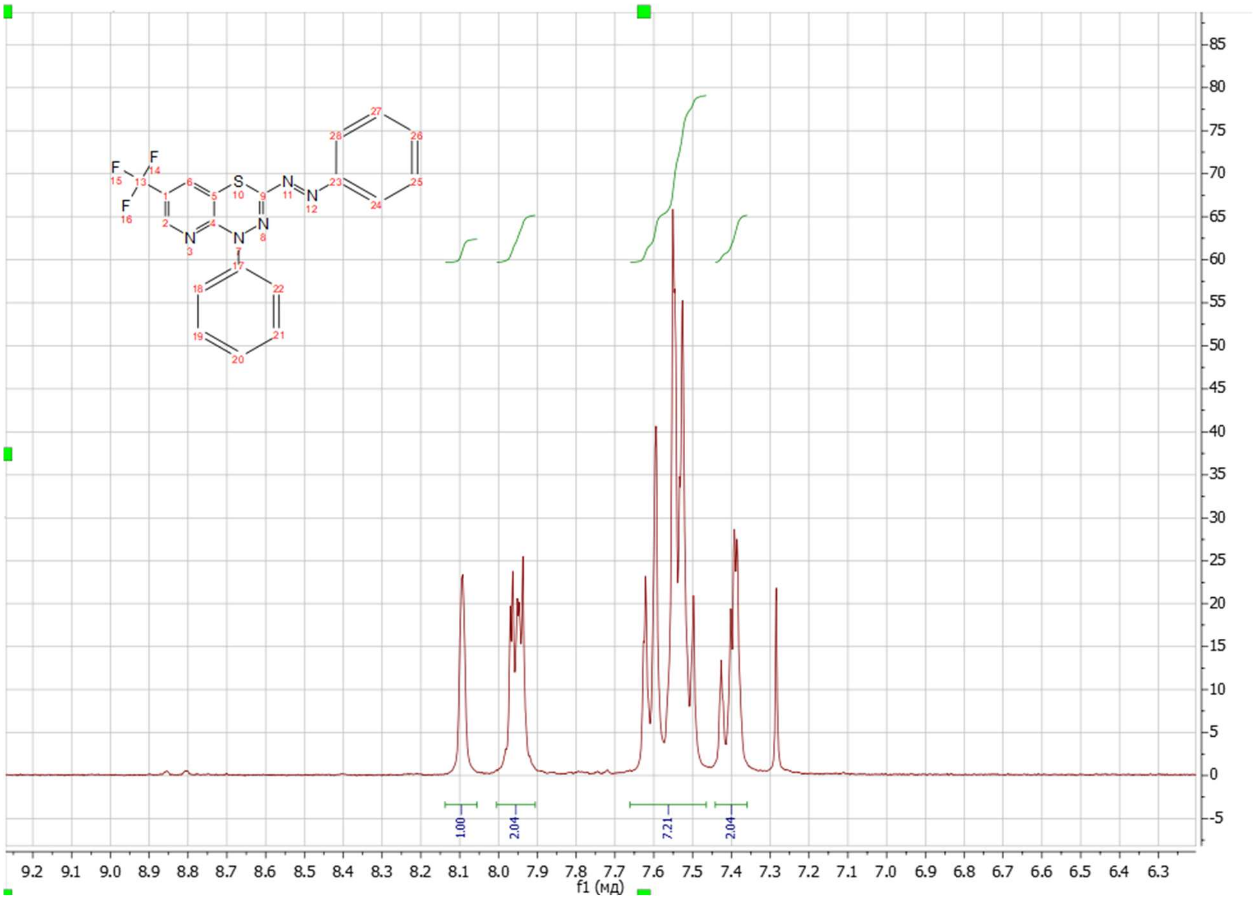
1.00
2.04
7.21
2.04
f1 (мд)

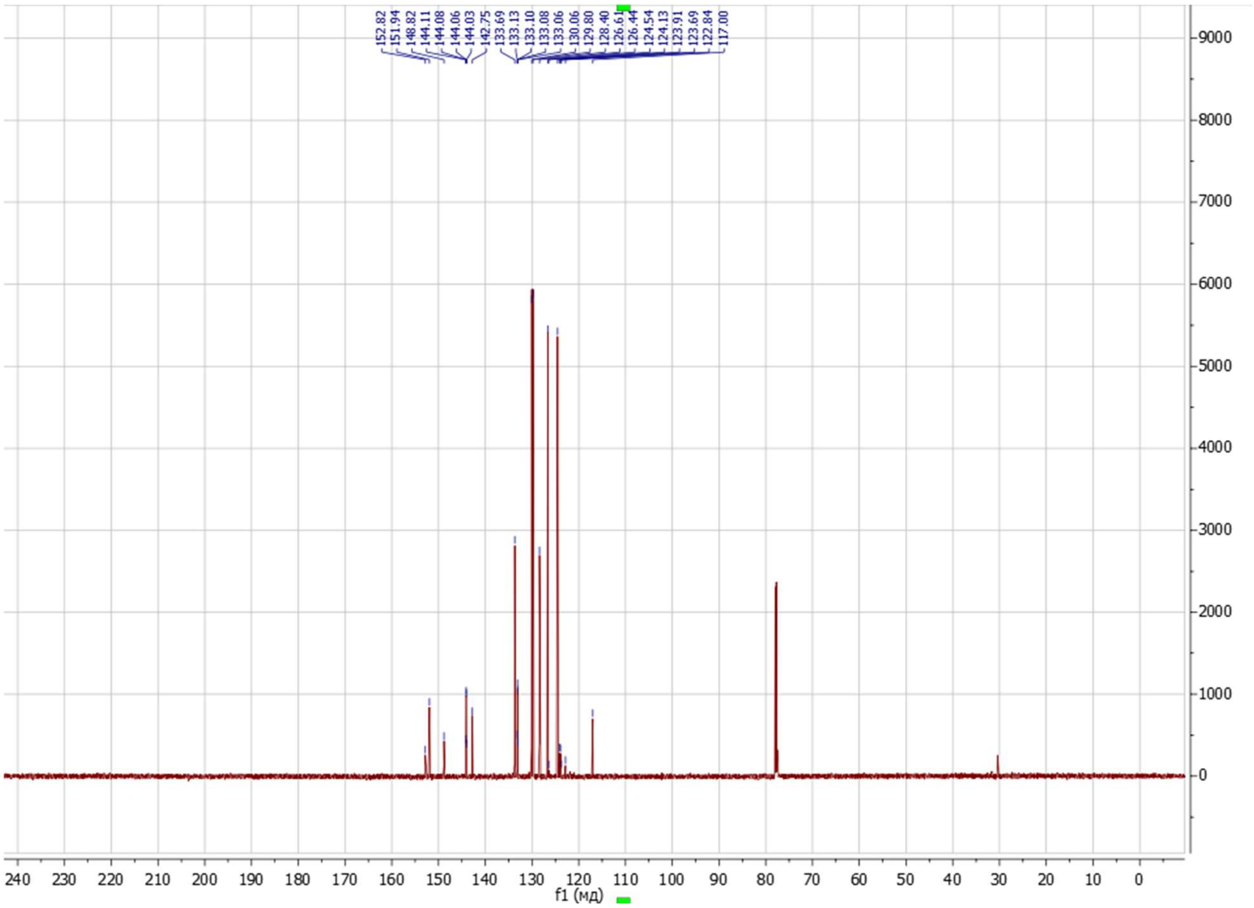
152.82
151.94
148.82
144.11
144.08
144.06
144.03
142.75
133.69
133.13
133.10
133.08
133.06
130.06
129.80
128.40
126.61
126.44
124.54
124.13
123.91
123.69
122.84
117.00
f1 (мд)

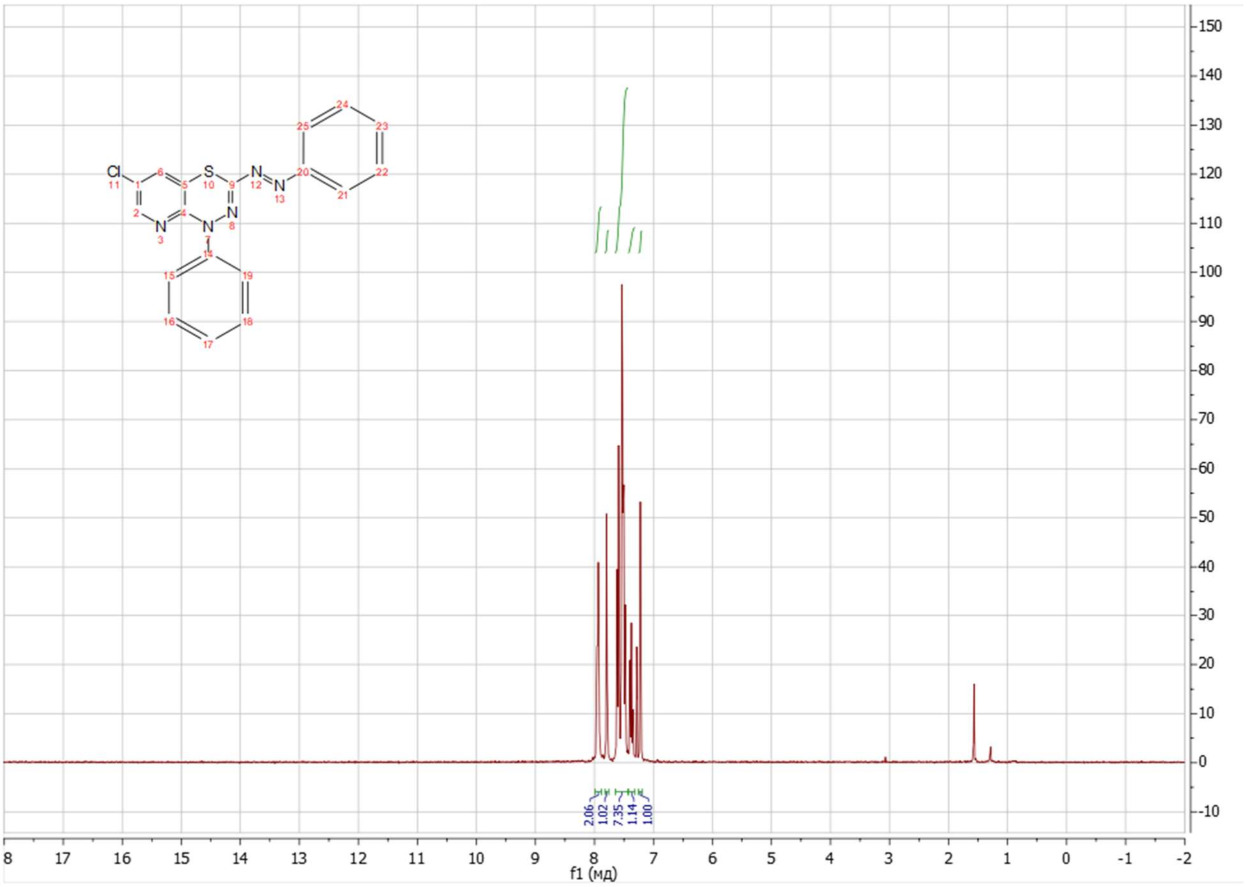
Cl
S
N
N
N
N
N
2.06
1.02
7.35
1.14
1.00
f1 (мд)

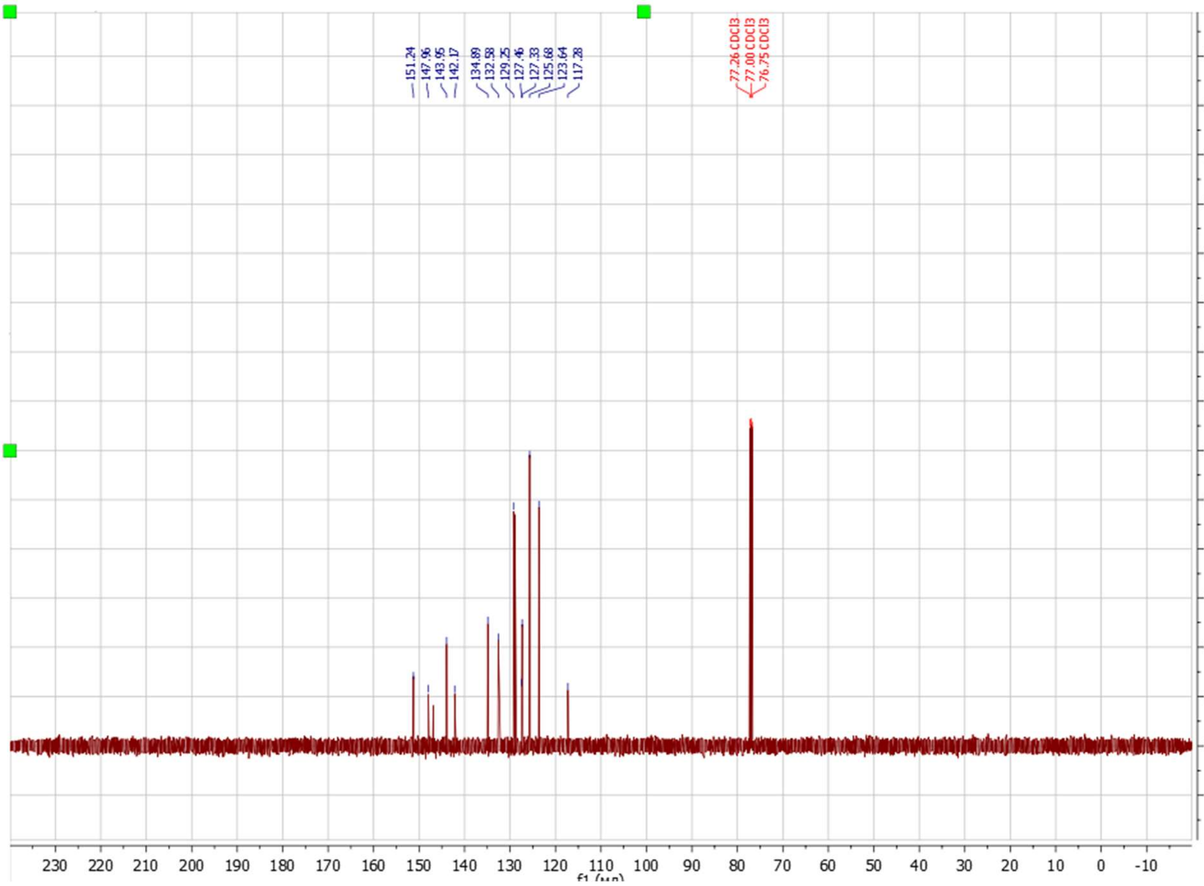
151.24
147.96
143.95
142.17
134.89
132.58
129.25
127.46
127.33
125.68
123.64
117.28
77.26 CDCl3
77.00 CDCl3
76.75 CDCl3
f1 (мд)

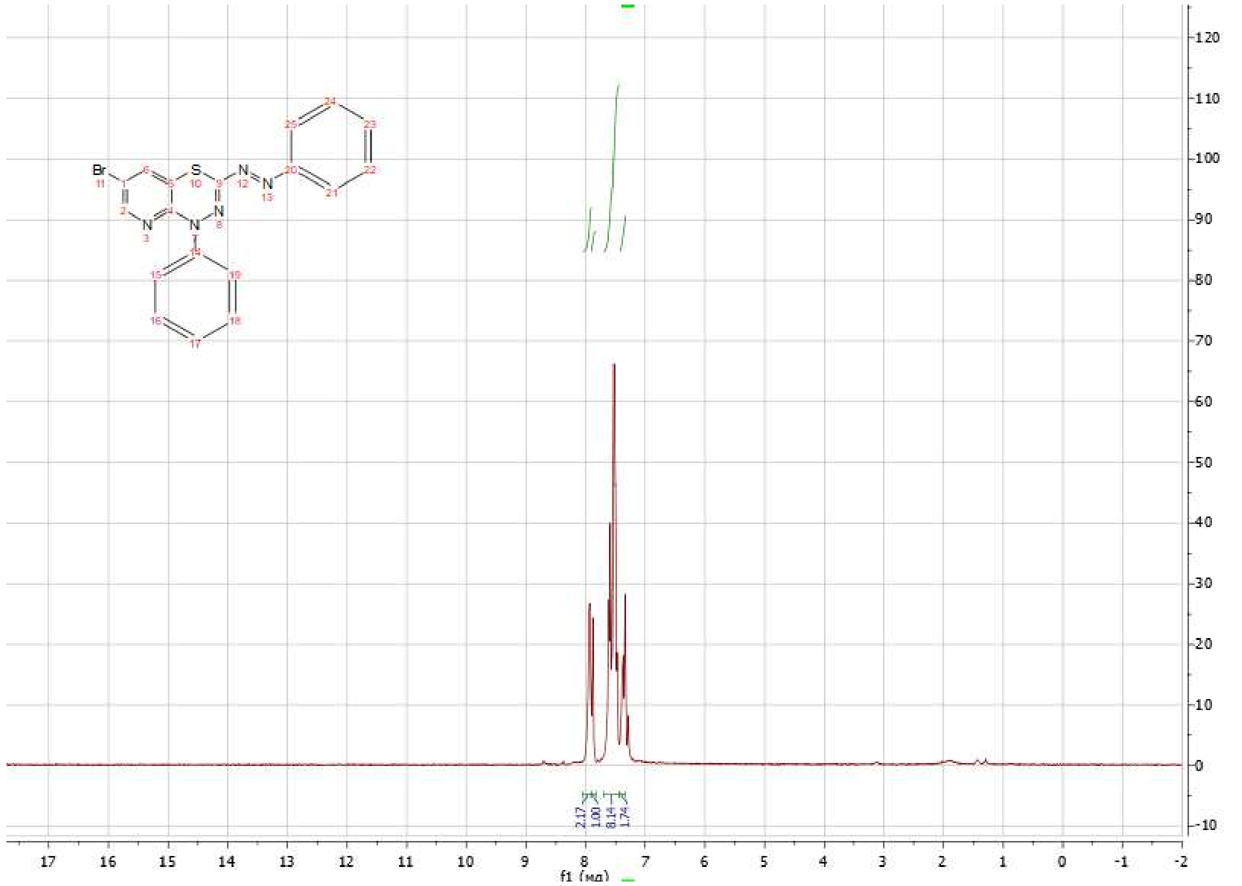
Br
S
N
N
N
N
N
2.17
1.00
8.14
1.74

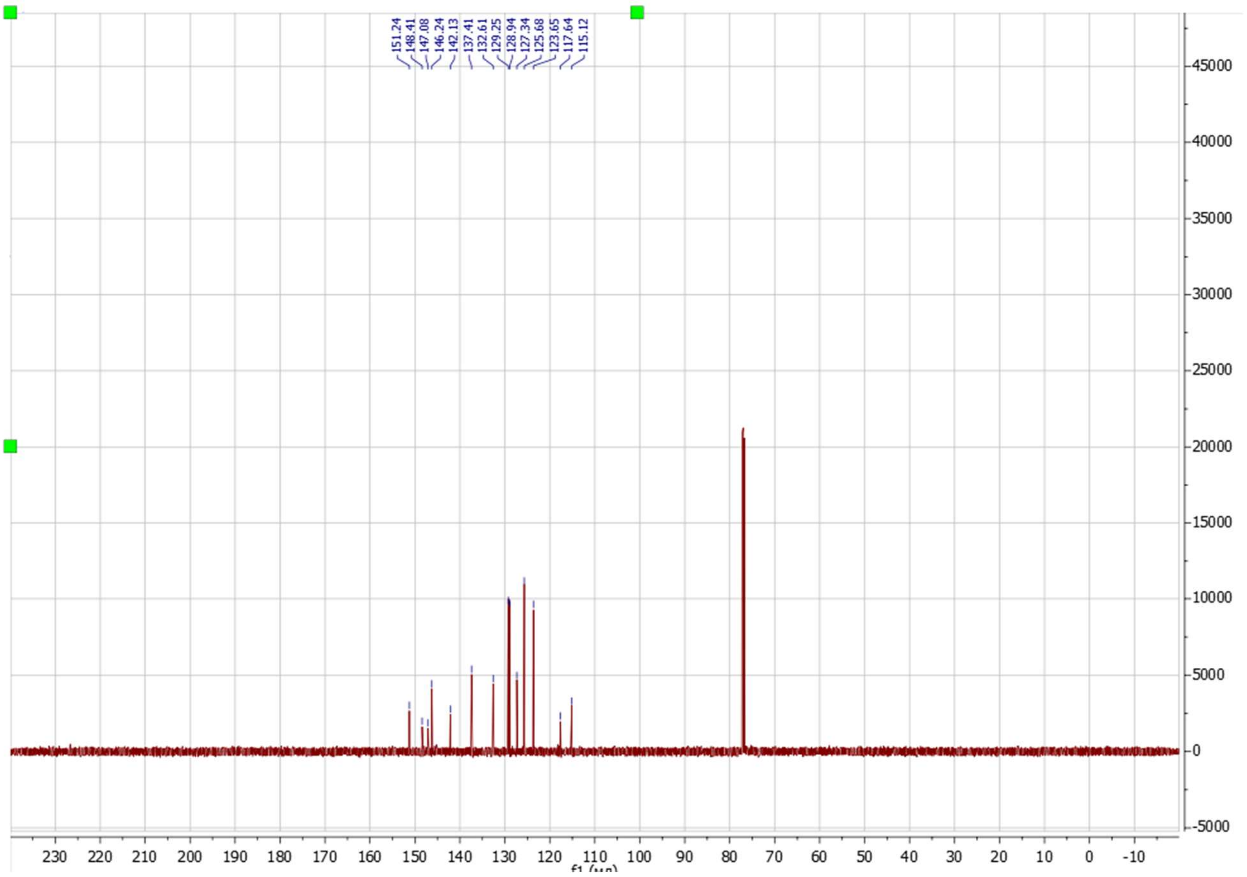
151.24
148.41
147.08
146.24
142.13
137.41
132.61
129.25
128.94
127.34
125.68
123.65
117.64
115.12

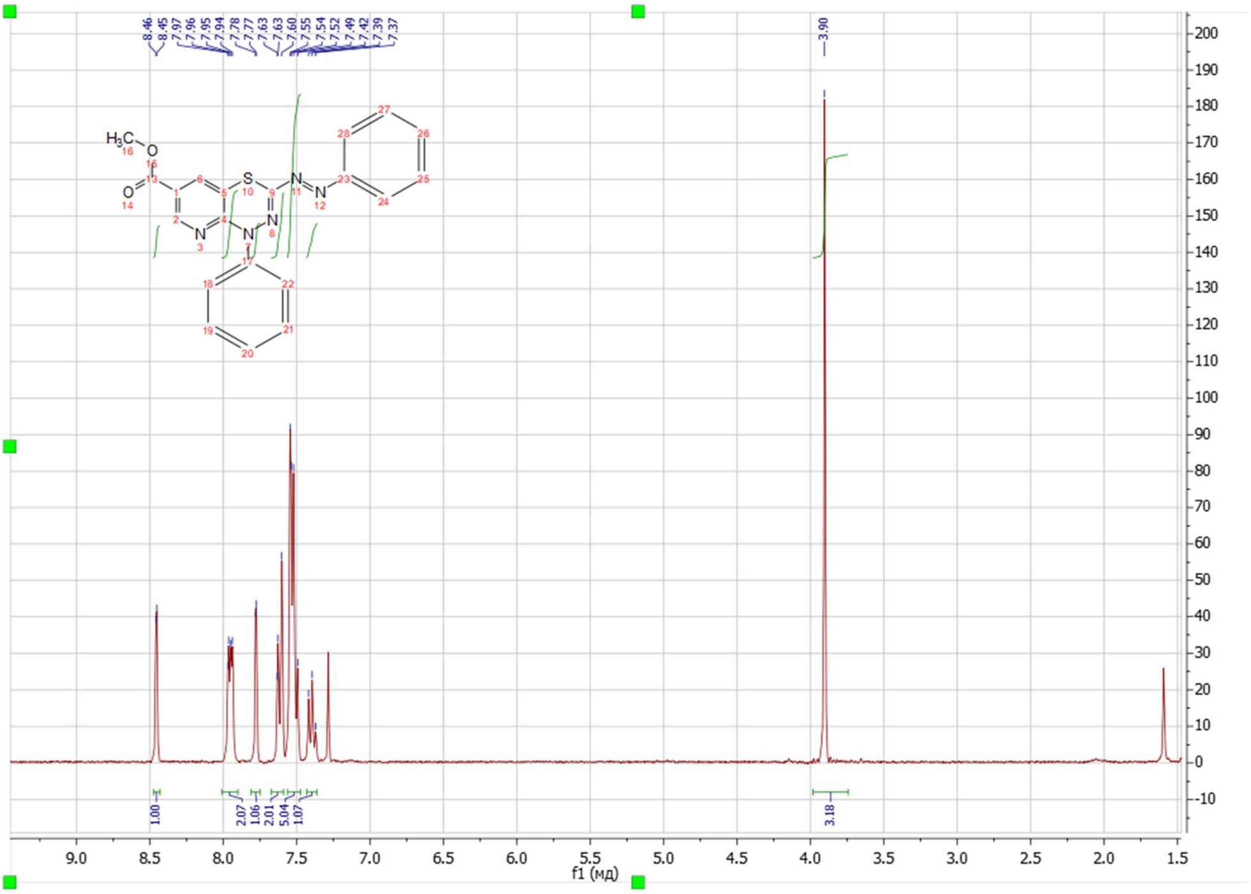
8.46 8.45 7.97 7.95 7.96 7.94 7.78 7.77 7.63 7.60 7.55 7.54 7.52 7.49 7.42 7.39 7.37
3.90
H3C O O S N N N N N
1.00 2.07 1.06 2.01 5.04 1.07
3.18
f1 (мд)

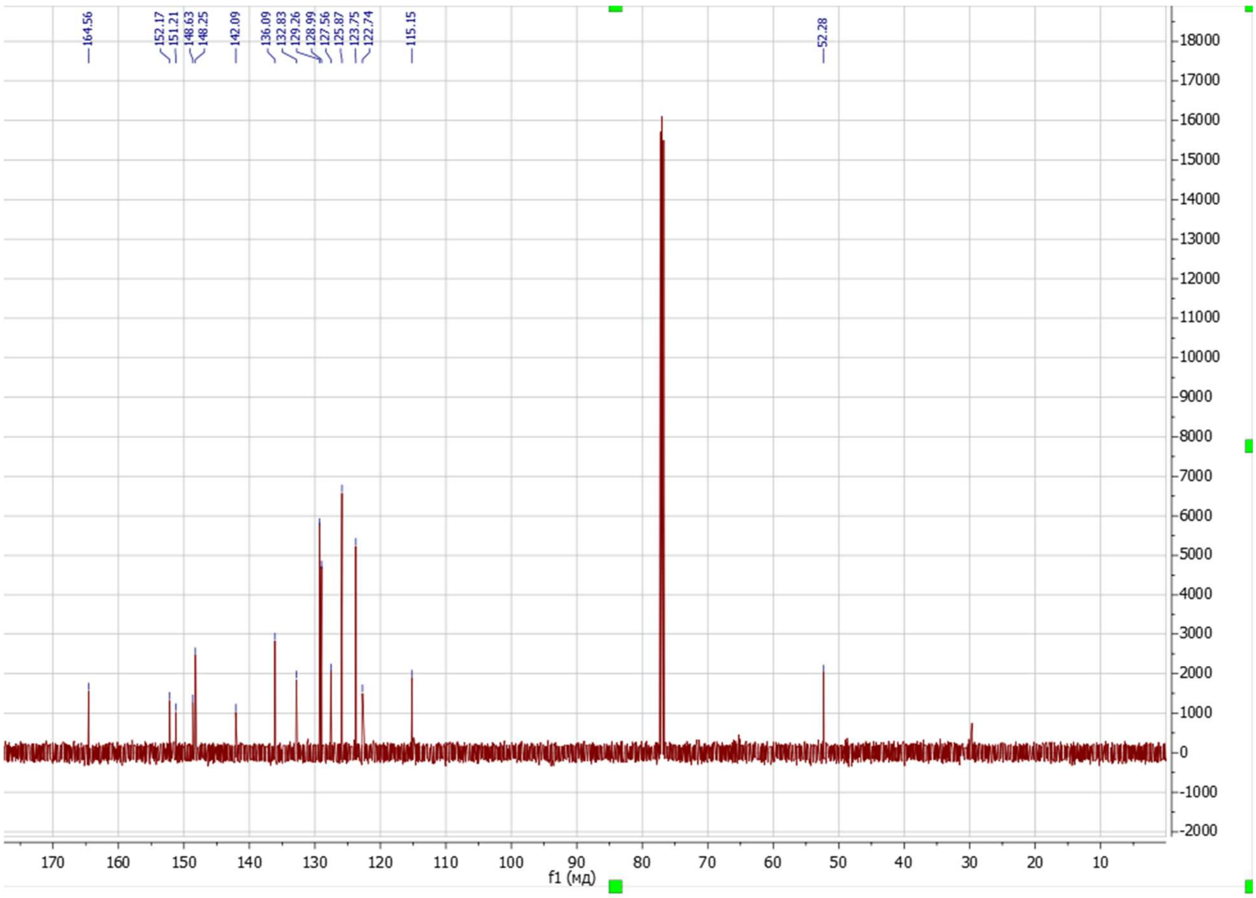
164.56
152.17 151.21 148.63 148.25
142.09
136.09 132.83 129.26 128.99 127.56 125.87 123.75 122.74
115.15
52.28
f1 (мд)

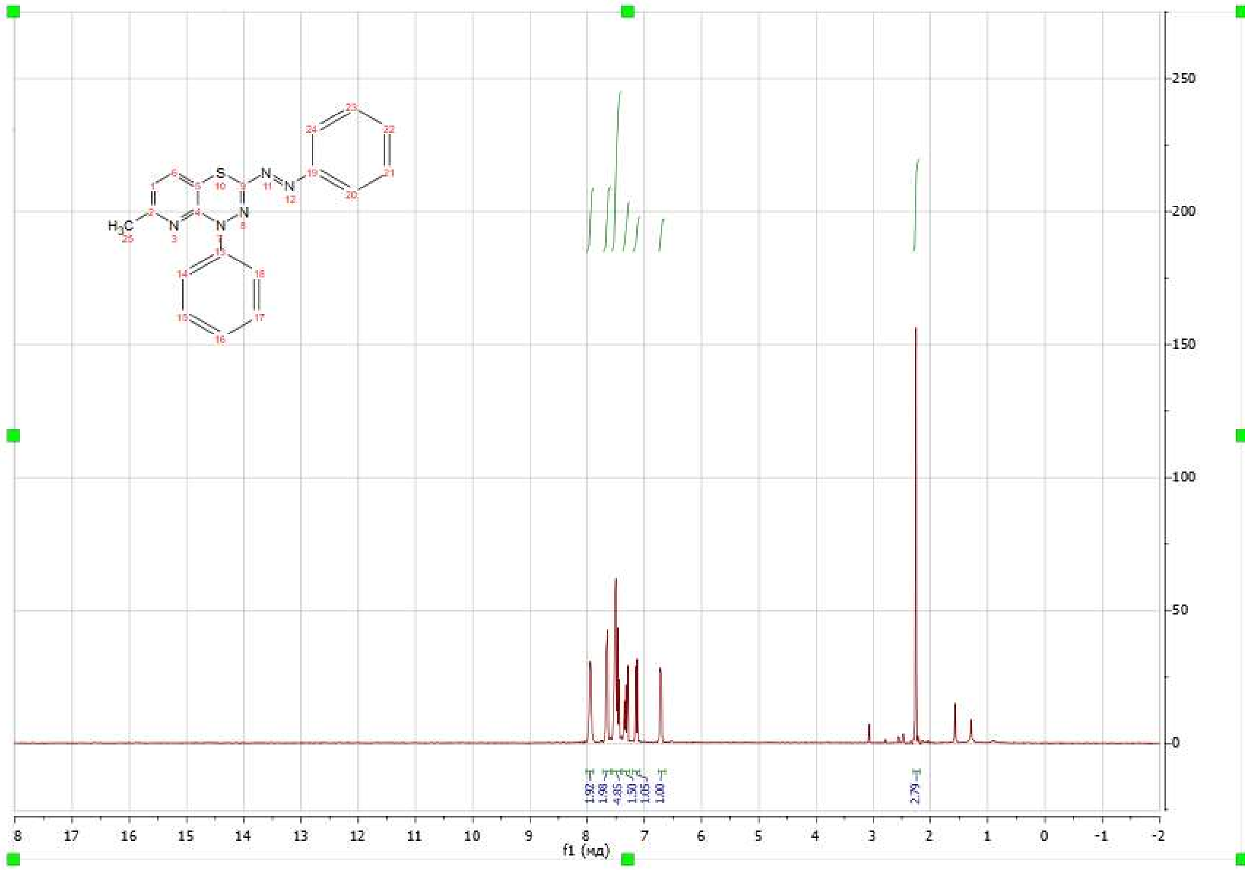

S
N
N
N
N
N
H_3C
1.92
1.98
4.85
1.50
1.05
1.00
2.79
f1 (мд)

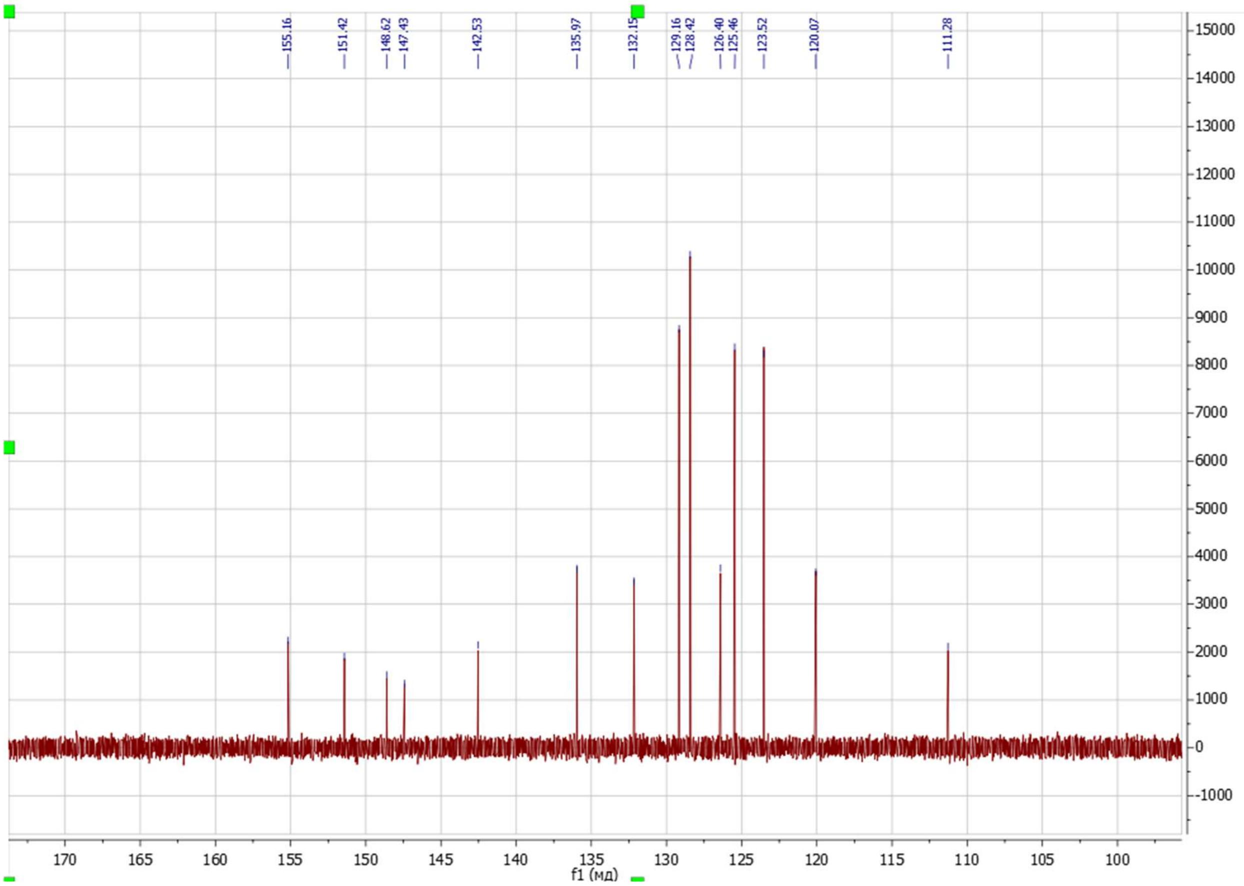

155.16
151.42
148.62
147.43
142.53
135.97
132.15
129.16
128.42
126.40
125.46
123.52
120.07
111.28
f1 (мд)

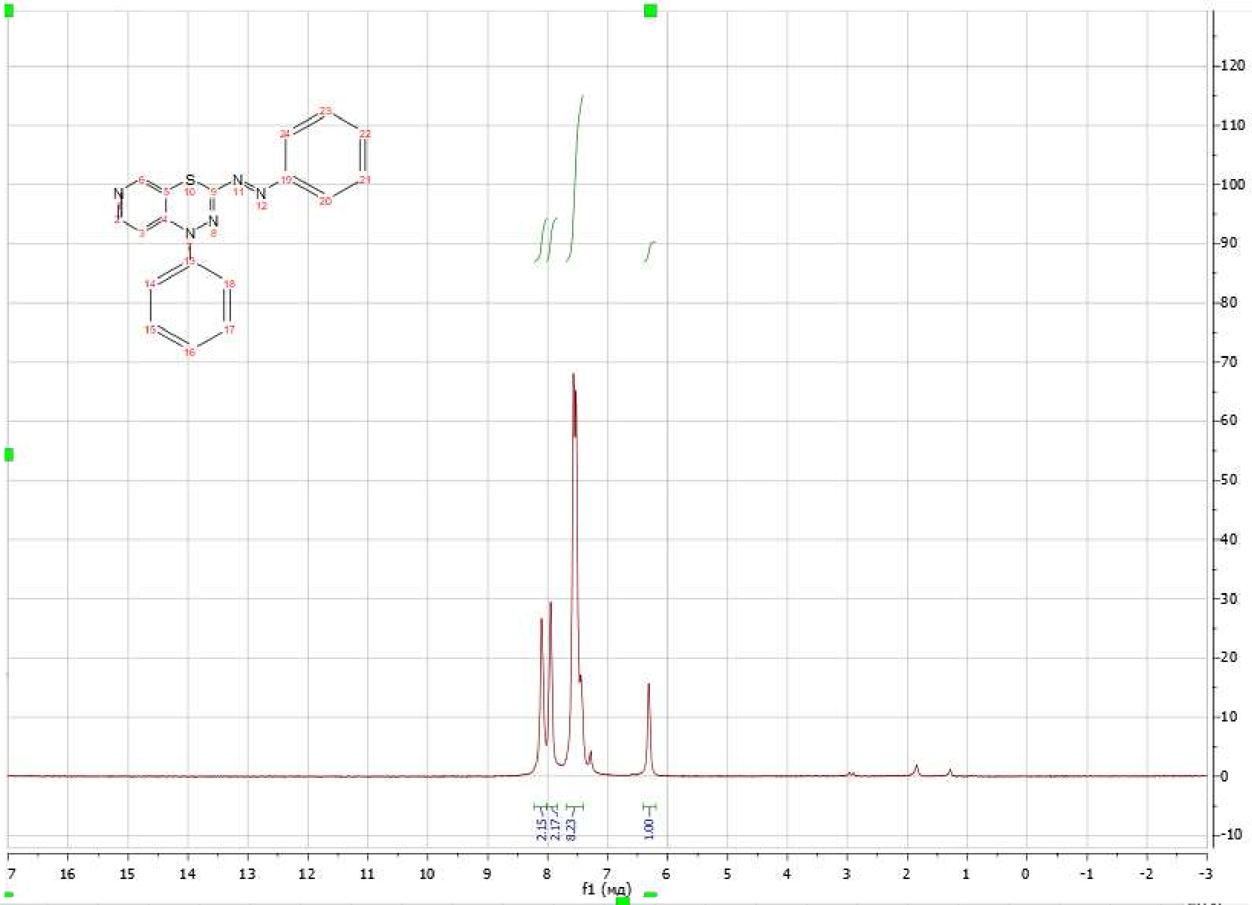
S
N
N
N
N
N
2.15
2.17
8.23
1.00
f1 (мд)

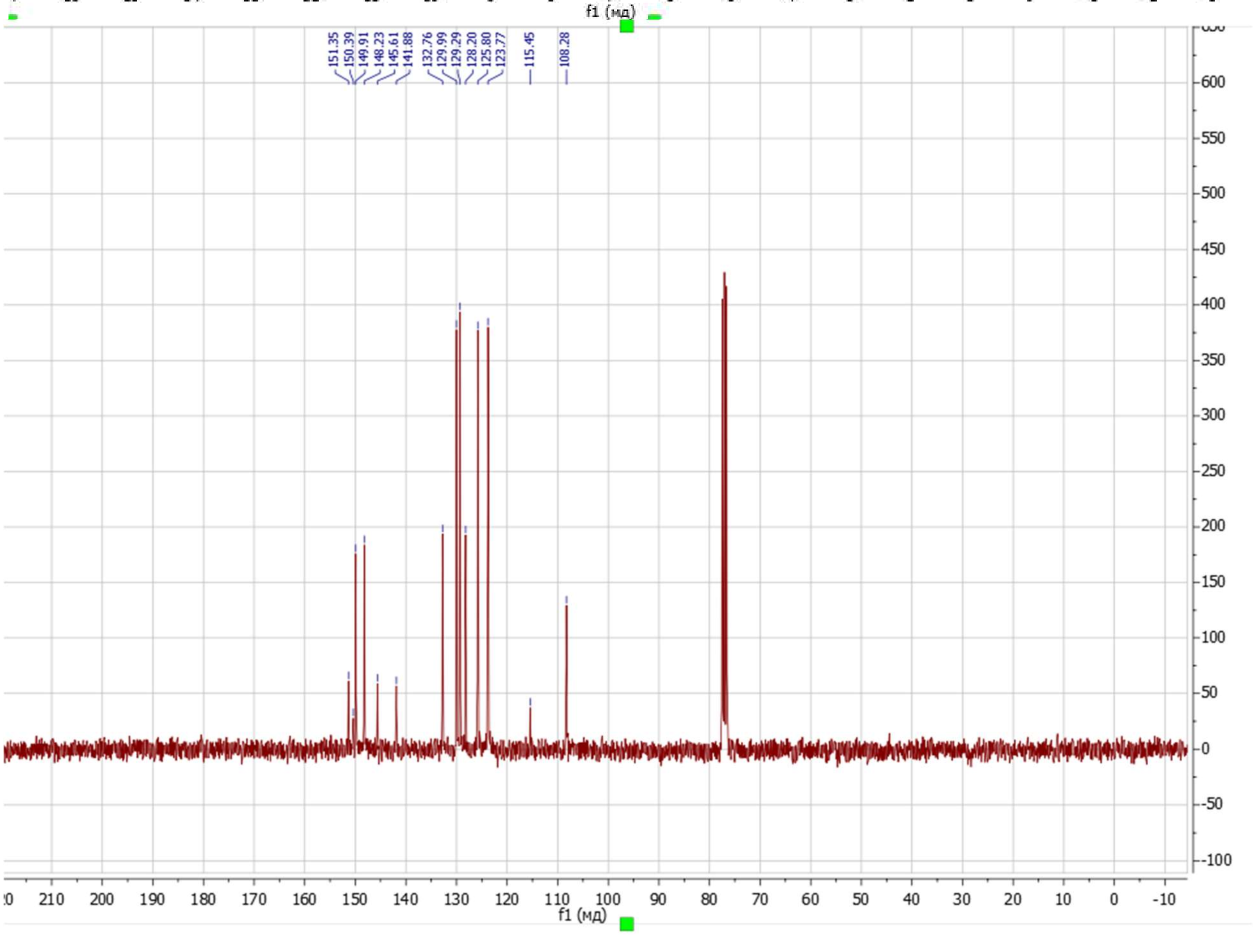
151.35
150.39
149.91
148.23
145.61
141.88
132.76
129.99
129.29
128.20
125.80
123.77
115.45
108.28
f1 (мд)

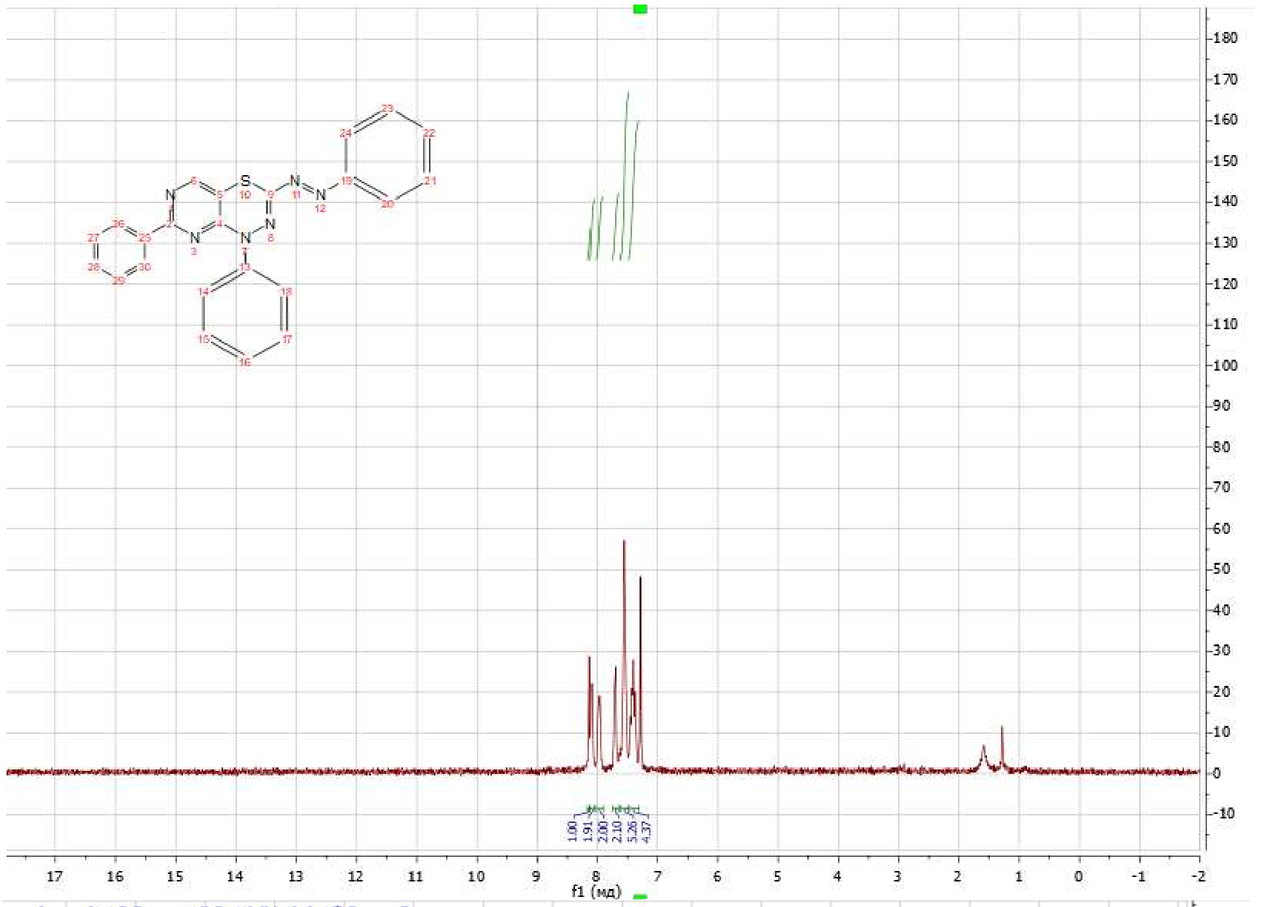
1.00
1.91
2.00
2.10
5.26
4.37
f1 (мд)

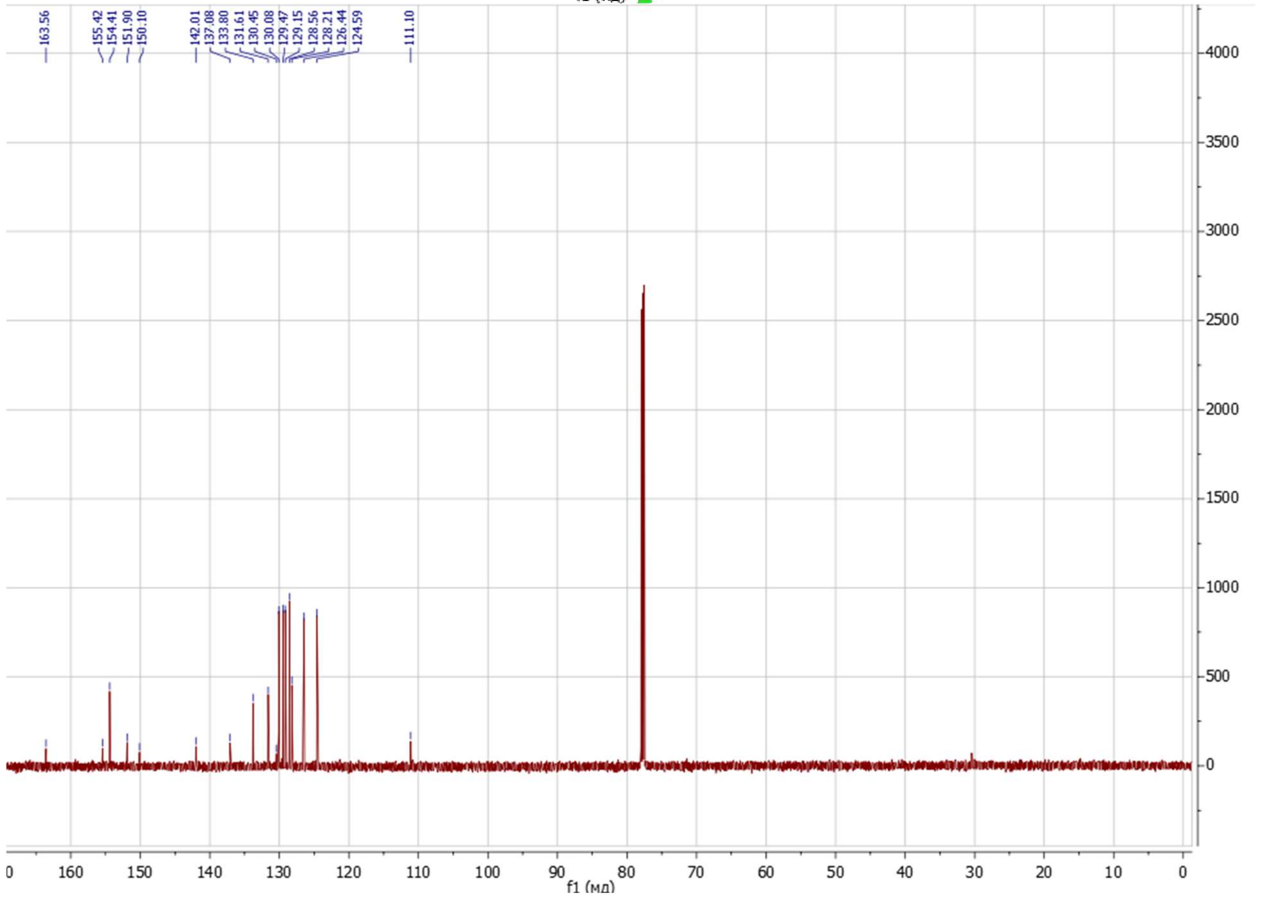
163.56
155.42
154.41
151.90
150.10
142.01
137.08
133.80
131.61
130.45
130.08
129.47
129.15
128.56
128.21
126.44
124.59
111.10
f1 (мд)

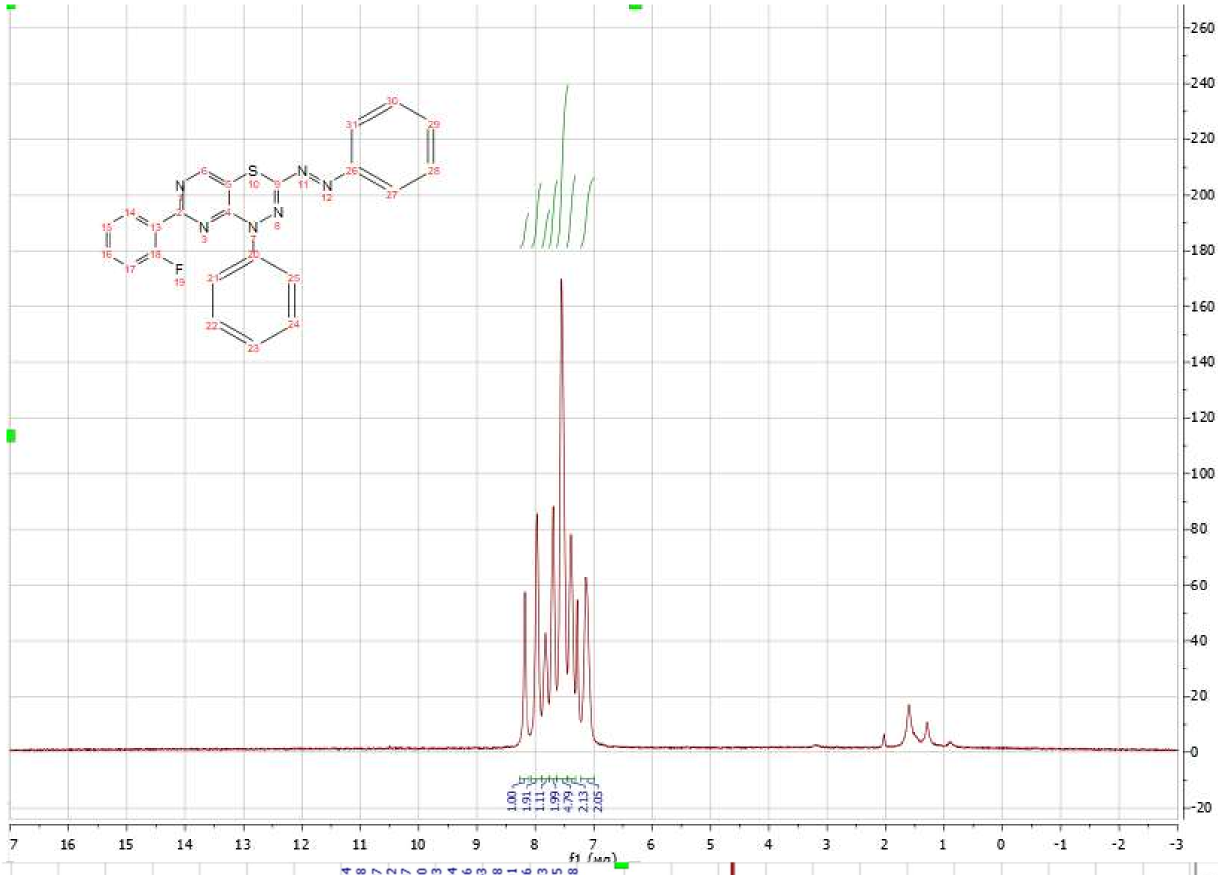
1.00
1.91
1.11
1.99
4.79
2.13
2.05
f1 (мд)

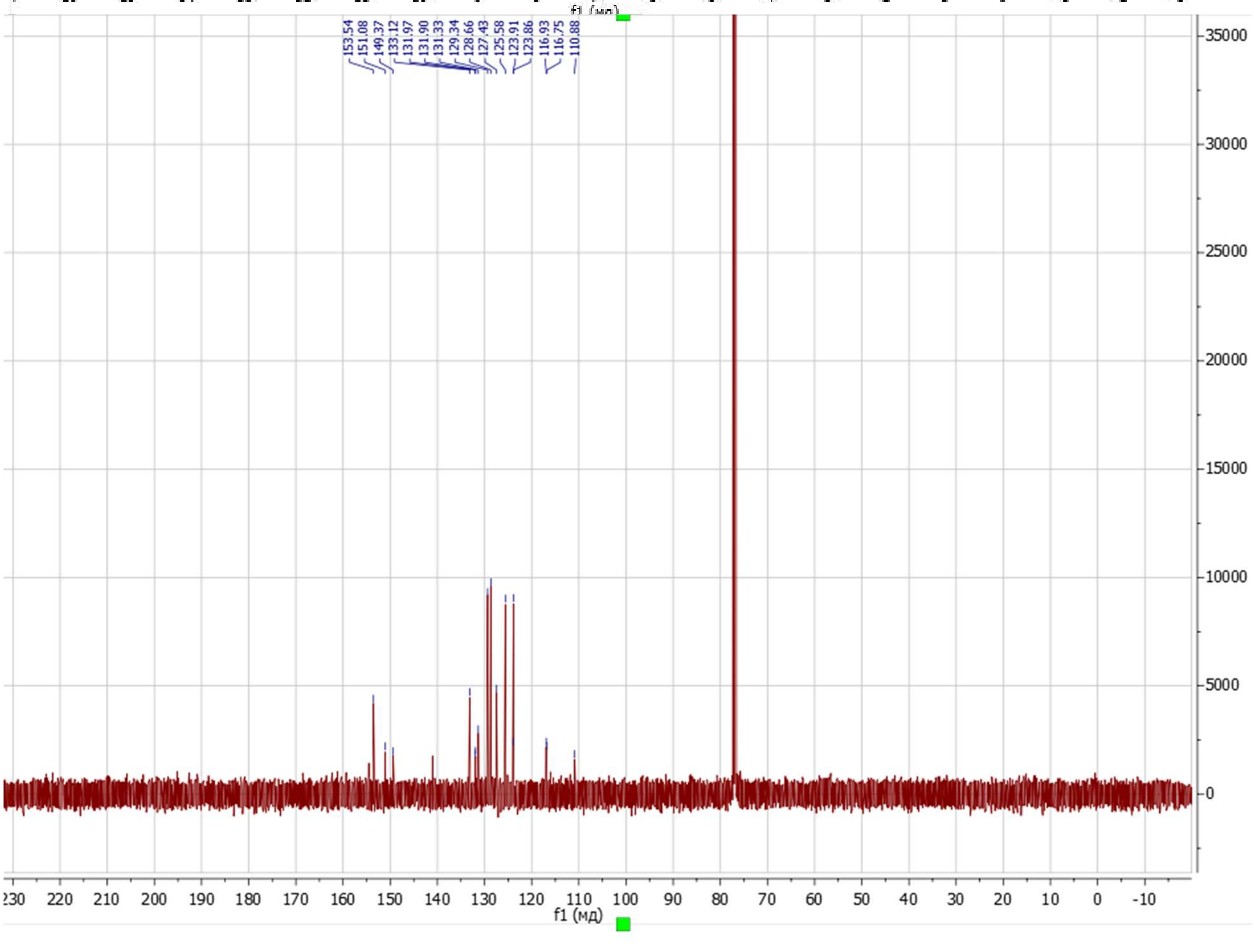
153.54
151.08
149.37
133.12
131.97
131.90
131.33
129.34
128.66
127.43
125.58
123.91
123.86
116.93
116.75
110.88
f1 (мд)

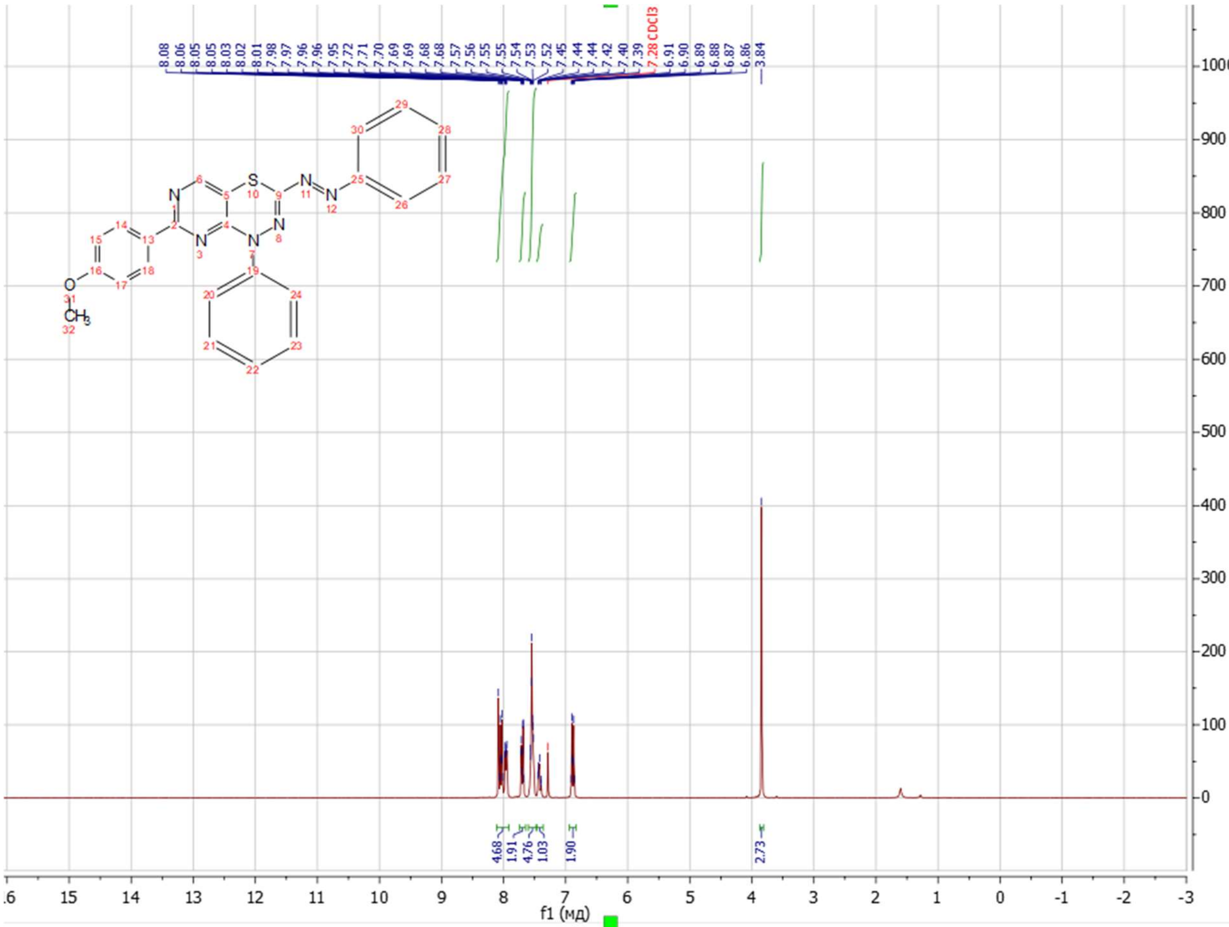
8.08 8.06 8.05 8.03 8.02 8.01 7.98 7.97 7.96 7.96 7.95 7.72 7.71 7.70 7.69 7.69 7.68 7.68 7.57 7.56 7.55 7.55 7.54 7.53 7.52 7.45 7.44 7.44 7.42 7.40 7.39
7.28 CDCl3
6.91 6.89 6.88 6.87 6.86
3.84
4.68 1.91 4.76 1.03 1.90 2.73
f1 (мд)

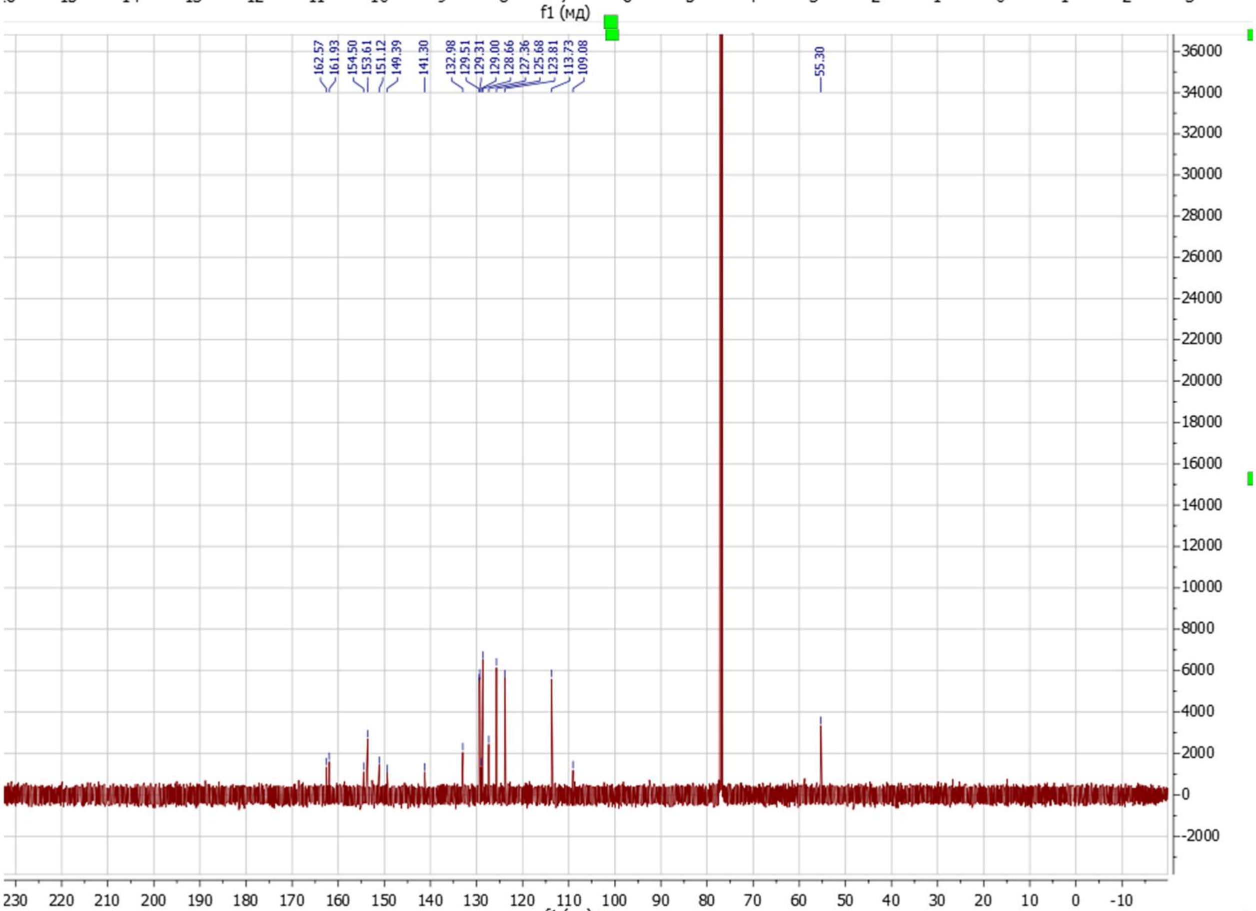
162.57 161.93 154.50 153.61 151.12 149.39 141.30 132.98 129.51 129.31 129.00 128.66 127.36 125.68 123.81 113.73 109.08
55.30
f1 (мд)

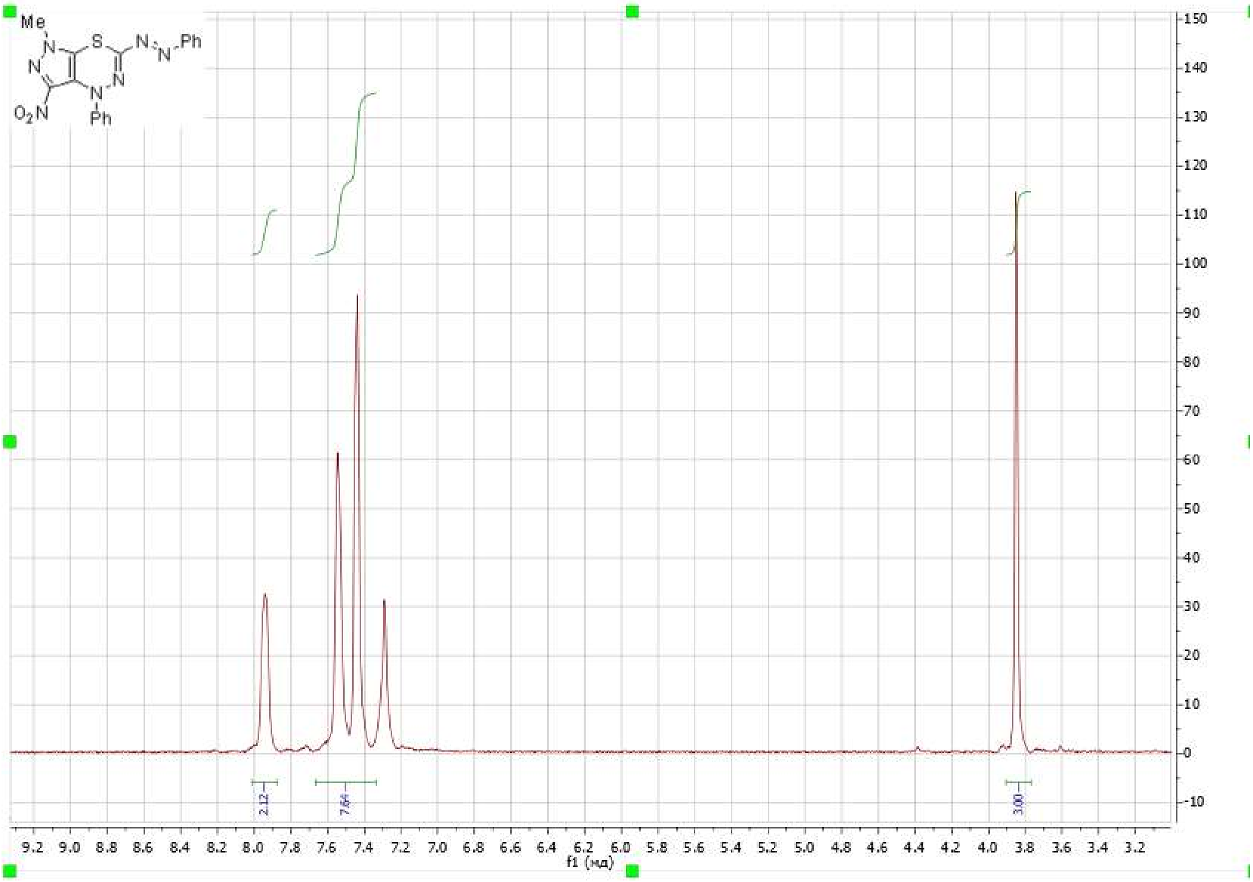

Me
S
N
N
N
Ph
O2N
Ph
2.12
7.64
3.00
f1 (мд)

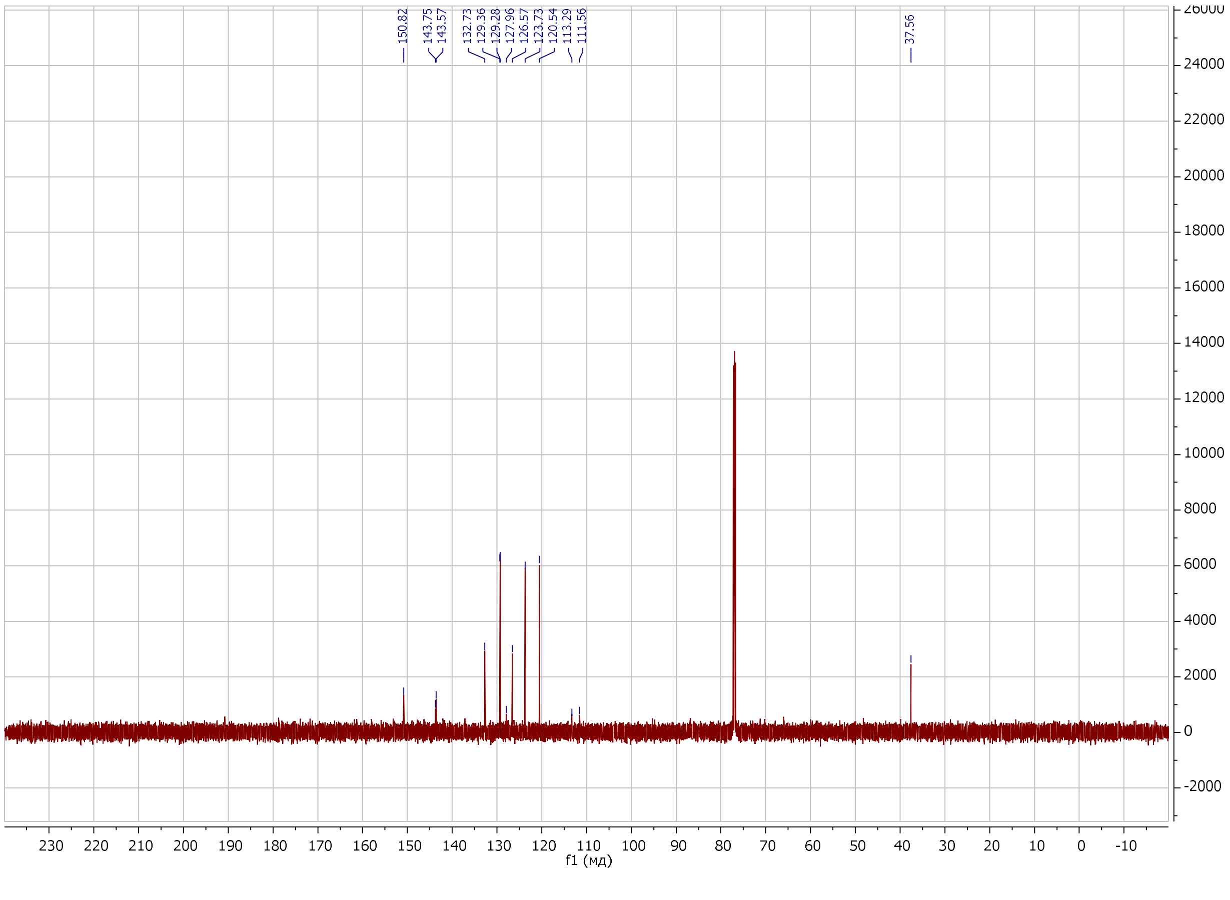

150.82
143.75
143.57
132.73
129.36
129.28
127.96
126.57
123.73
120.54
113.29
111.56
37.56
f1 (мд)

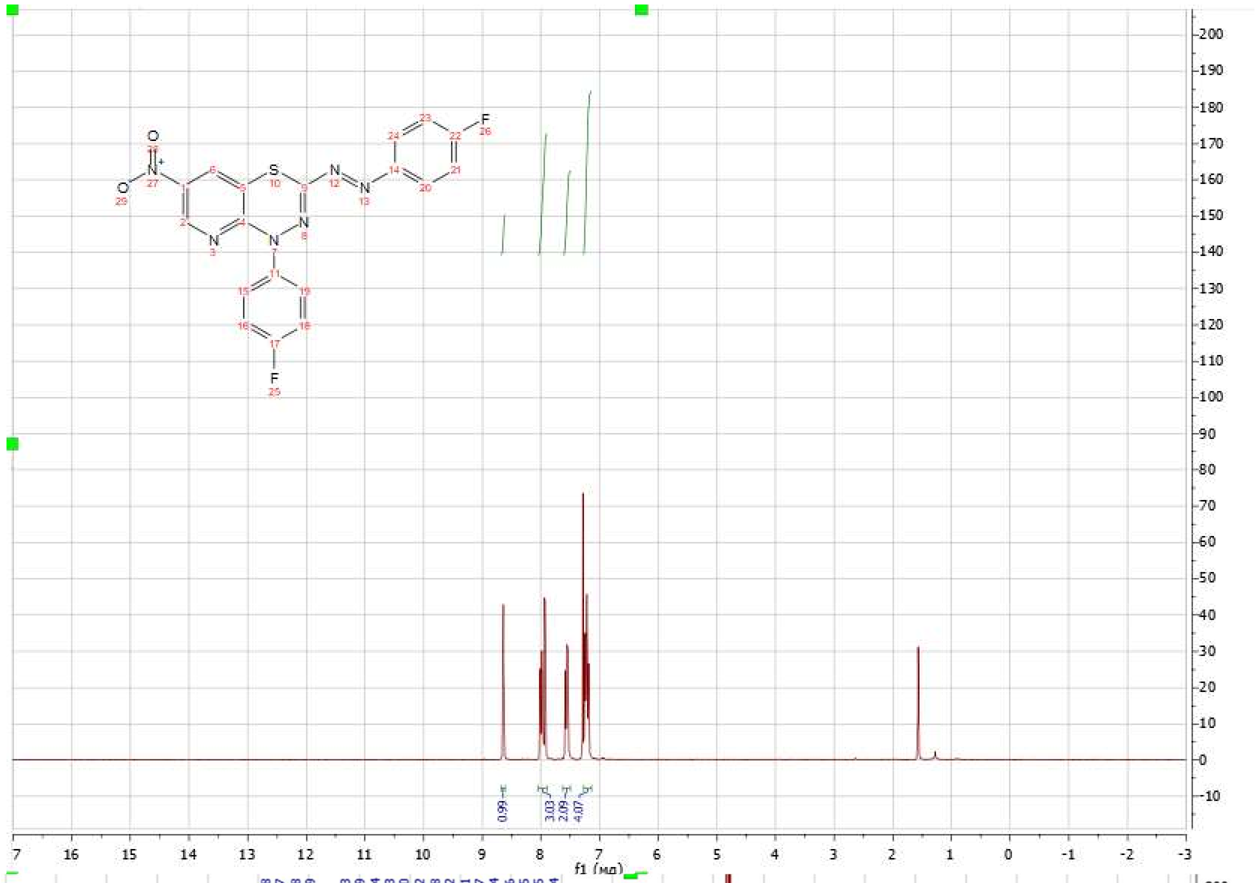

S
N
F
O
0.99
3.03
2.09
4.07

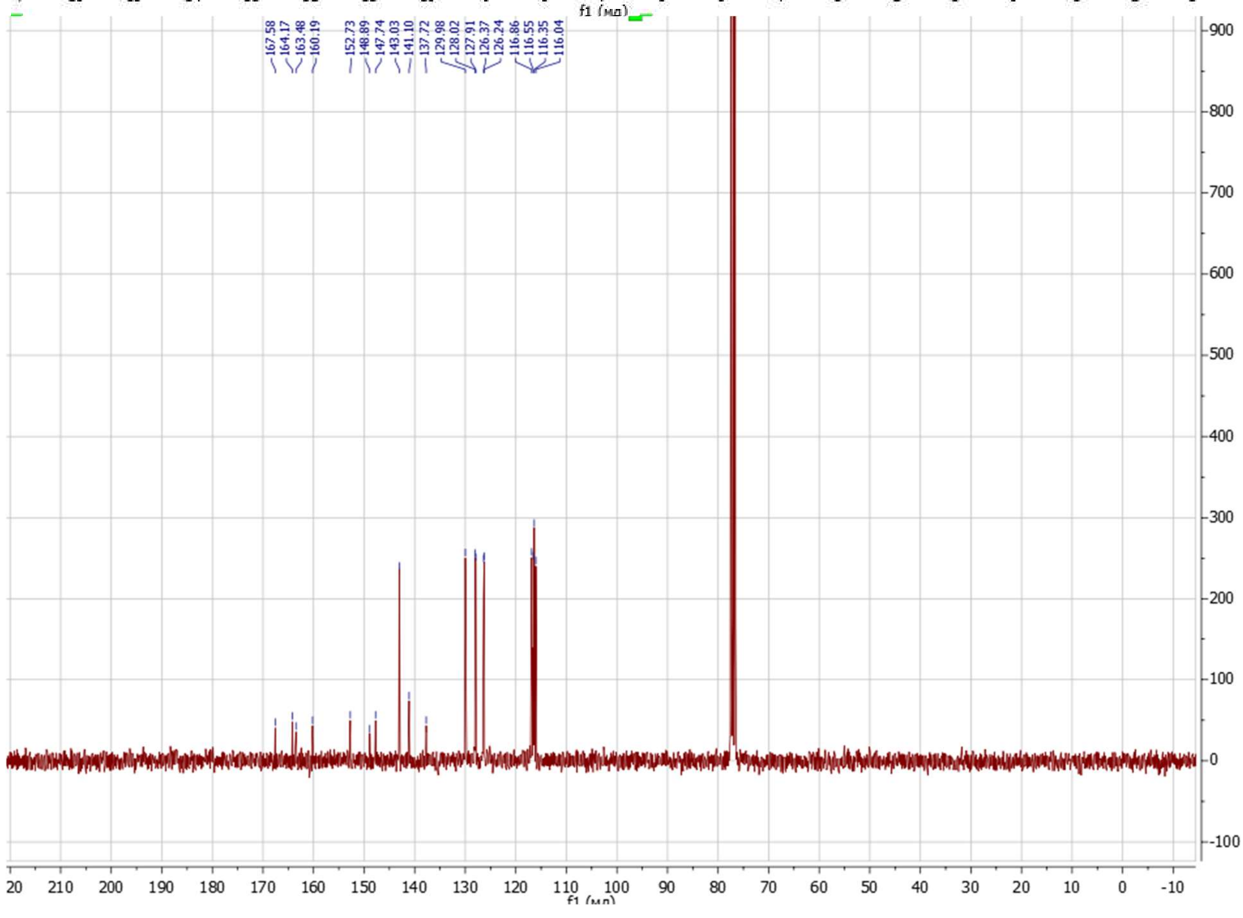

167.58
164.17
163.48
160.19
152.73
148.89
147.74
143.03
141.10
137.72
129.98
128.02
127.91
126.37
126.24
116.86
116.55
116.35
116.04

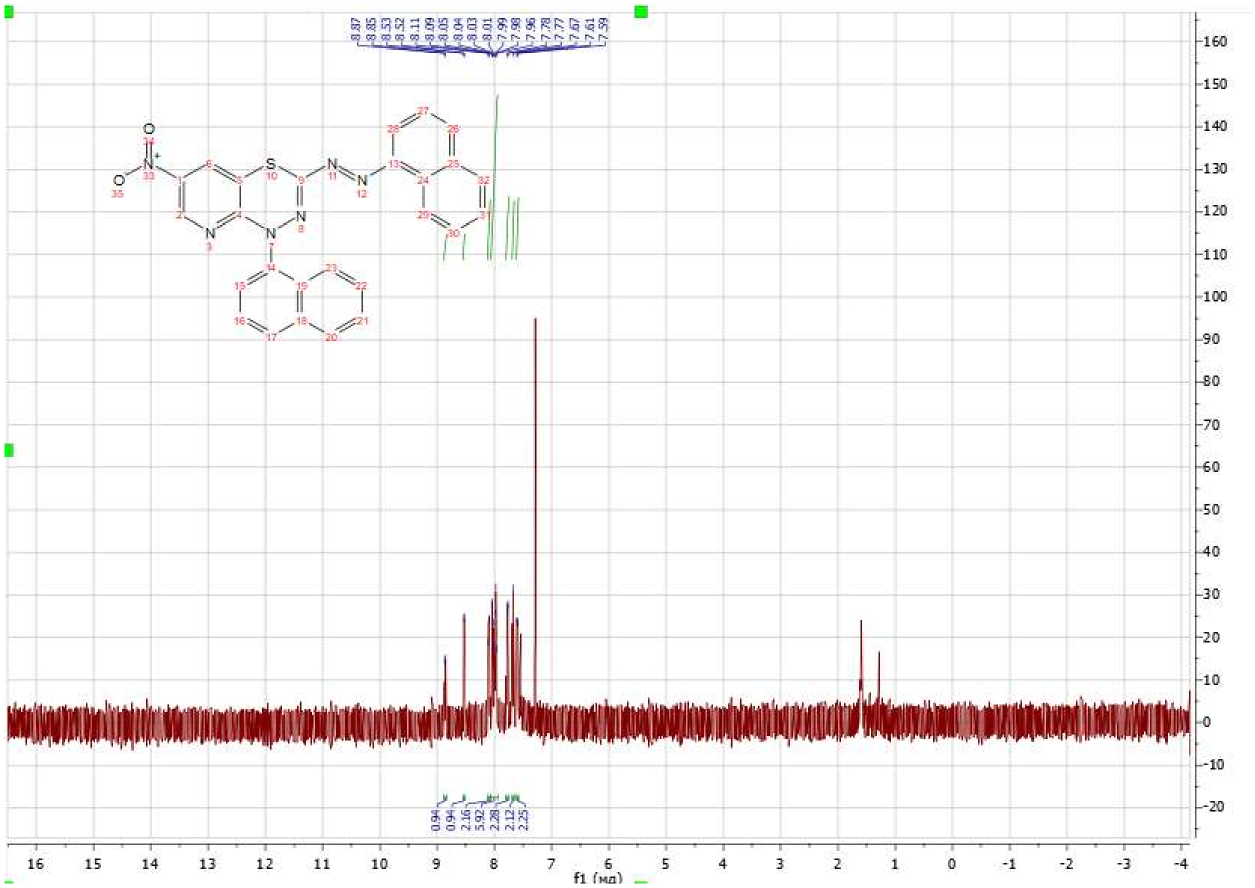
8.87 8.85 8.53 8.52 8.11 8.09 8.05 8.04 8.03 8.01 7.99 7.98 7.96 7.78 7.77 7.67 7.61 7.59
0.94 0.94 2.16 5.92 2.28 2.12 2.25
f1 (мд)

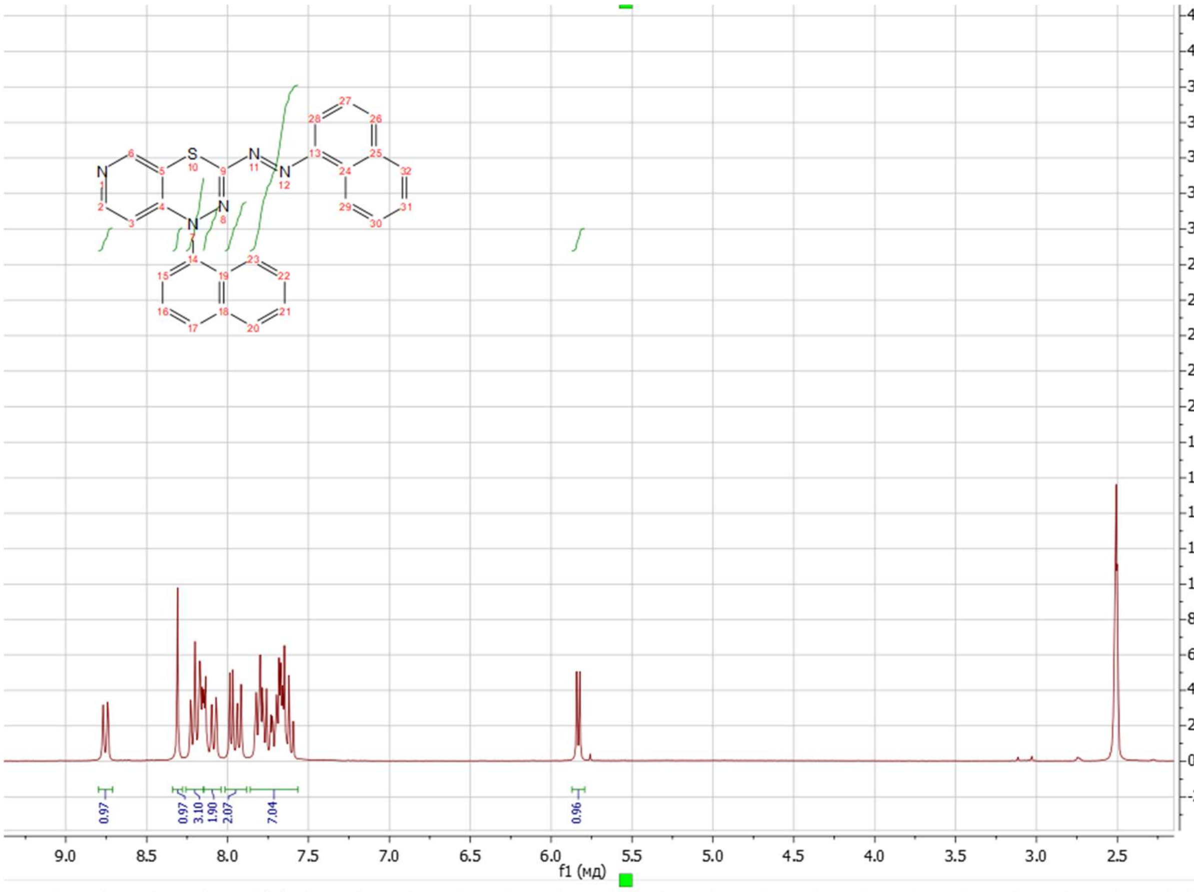
0.97
0.97
3.10
1.90
2.07
7.04
0.96
f1 (мд)

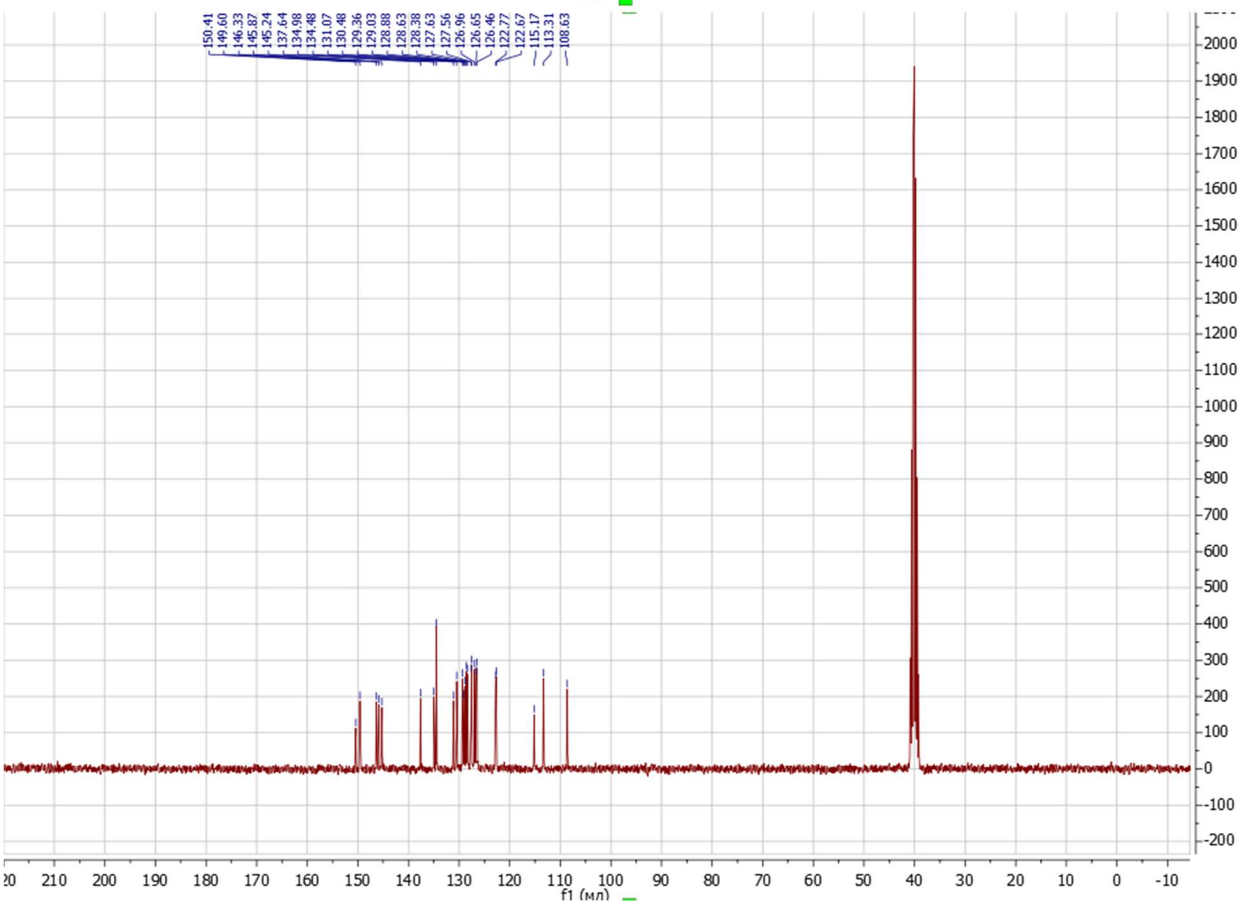
150.41
149.60
146.33
145.87
145.24
137.64
134.98
134.48
131.07
130.48
129.36
129.03
128.88
128.63
128.38
127.63
127.56
126.96
126.65
126.46
122.77
122.67
115.17
113.31
108.63
f1 (мд)

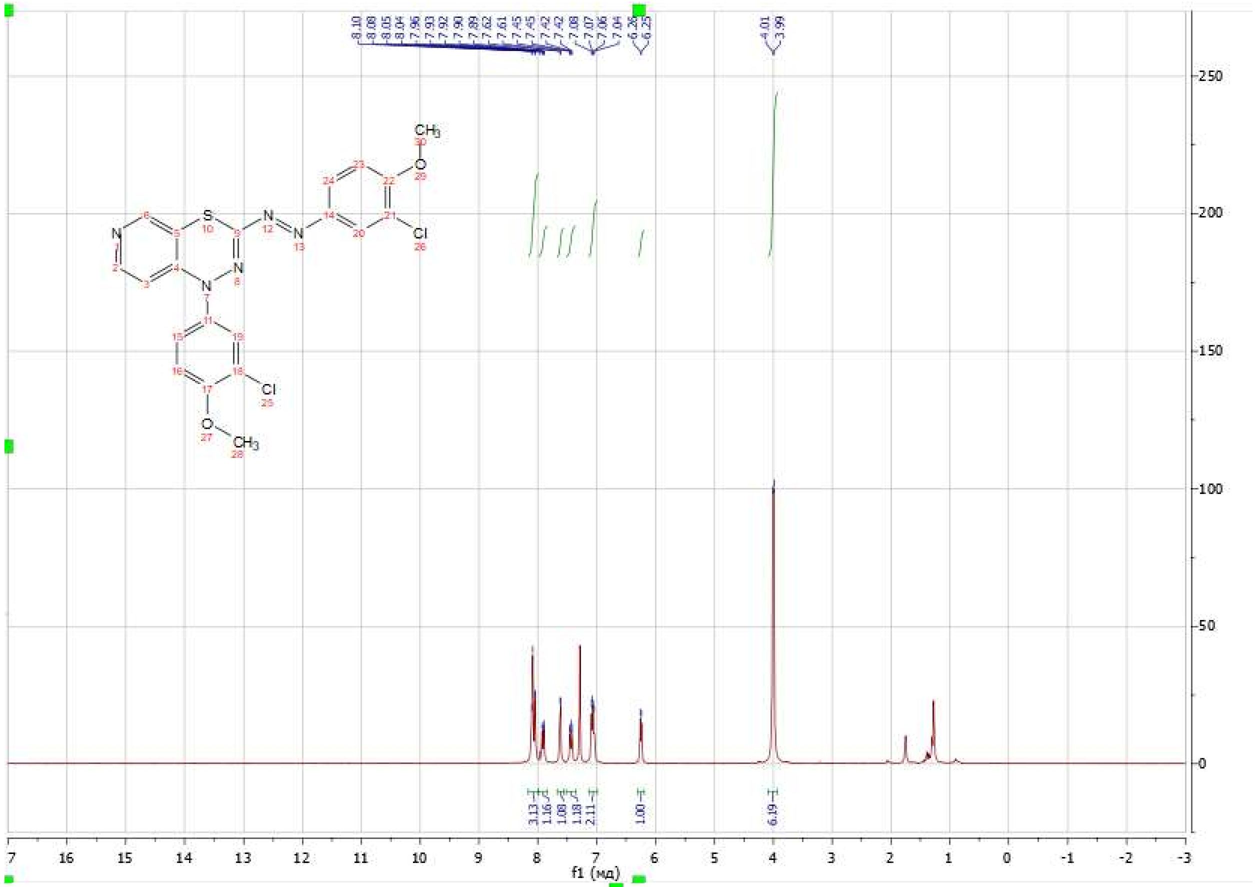
8.10 8.08 8.05 8.04 7.96 7.93 7.92 7.90 7.89 7.62 7.61 7.45 7.45 7.42 7.42 7.08 7.07 7.06 7.04 6.26 6.25
4.01 3.99
3.13 1.16 1.08 1.18 2.11 1.00 6.19
f1 (мд)

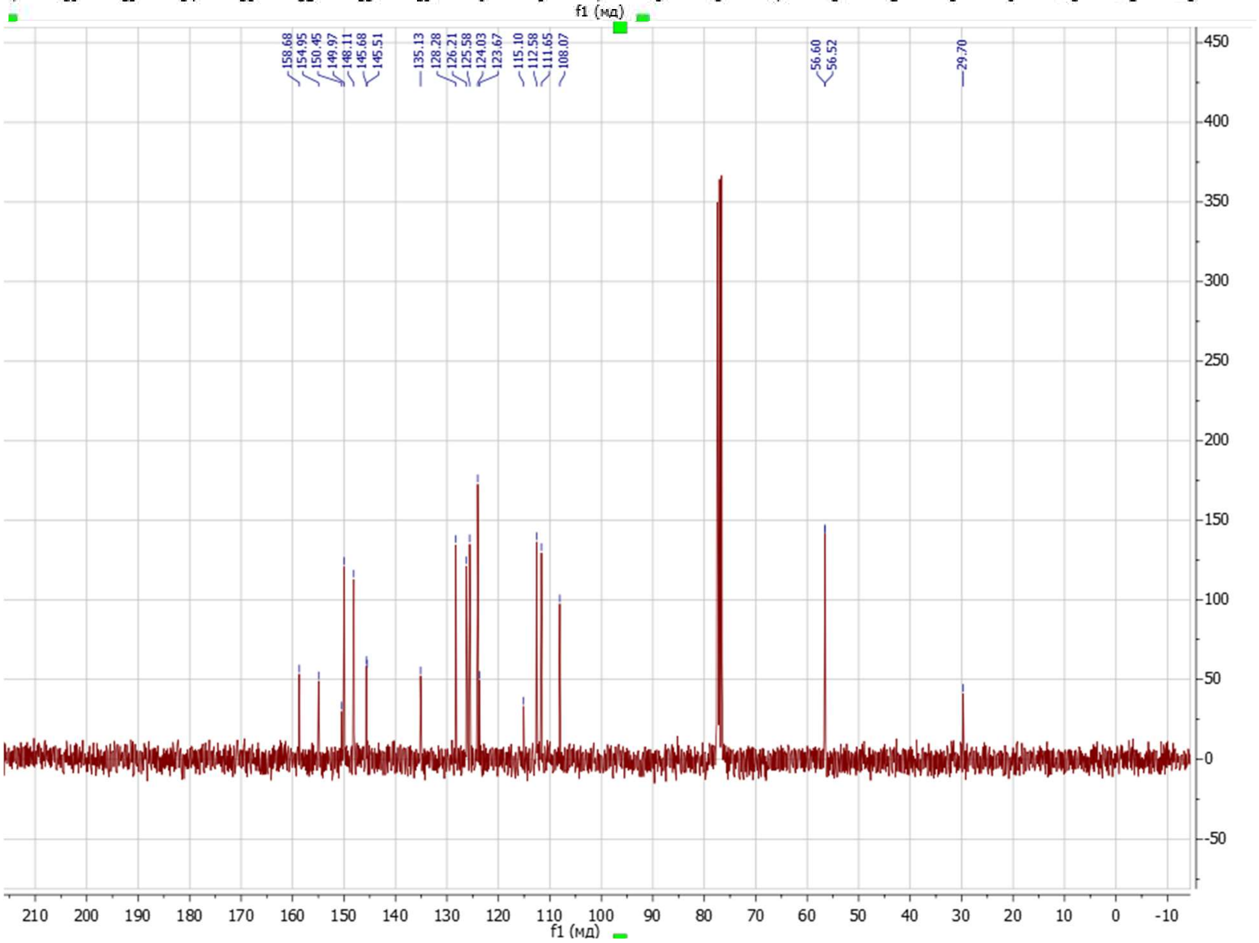
158.68 154.95 150.45 149.97 148.11 145.68 145.51
135.13 128.28 126.21 125.58 124.03 123.67
115.10 112.58 111.65 108.07
56.60 56.52
29.70
f1 (мд)

Table S1. Crystallographic data for **3a** and **4a**.

	**3a**	**4a**
CCDC	2580925	2580926
Formula	$C_{18}H_{12}N_6O_2S$	$C_{18}H_{10}F_2N_6O_2S$
Formula weight	376.40	412.38
T, K	120	120
Crystal system	monoclinic	monoclinic
Space group	$P2_1/n$	$C2/c$
Z / Z'	4 / 1	8 / 1
a, Å	4.5363(13)	28.5254(10)
b, Å	18.554(5)	10.9682(4)
c, Å	19.955(6)	11.7688(4)
β, °	91.429(7)	109.1197(9)
V, Å^3	1679.1(8)	3479.0(2)
d_{calc}, g cm^{-3}	1.489	1.575
μ, cm^{-1}	2.21	2.37
$2\theta_{max}$, °	52	60
Reflns. collected / independent	16559 / 3311	23224 / 5067
Observed reflections [I>2σ(I)]	2063	4185
R_1	0.0610	0.0315
wR_2	0.1529	0.0903
GOF	0.994	1.114
Residual density, e Å^{-3} (d_{max}/d_{min})	0.418/-0.325	0.326/-0.200

checkCIF/PLATON report

Structure factors have been supplied for datablock(s) bm1615

THIS REPORT IS FOR GUIDANCE ONLY. IF USED AS PART OF A REVIEW PROCEDURE FOR PUBLICATION, IT SHOULD NOT REPLACE THE EXPERTISE OF AN EXPERIENCED CRYSTALLOGRAPHIC REFEREE.

No syntax errors found. CIF dictionary Interpreting this report

Datablock: bm1615

Bond precision: C-C = 0.0046 A Wavelength=0.71073

Cell:	a=4.5363(13)	b=18.554(5)	c=19.955(6)
	alpha=90	beta=91.429(7)	gamma=90
Temperature:	120 K		

	Calculated	Reported
Volume	1679.0(8)	1679.1(8)
Space group	P 21/n	P 21/n
Hall group	-P 2yn	-P 2yn
Moiety formula	C18 H12 N6 O2 S	C18 H12 N6 O2 S
Sum formula	C18 H12 N6 O2 S	C18 H12 N6 O2 S
Mr	376.40	376.40
Dx,g cm-3	1.489	1.489
Z	4	4
Mu (mm-1)	0.221	0.221
F000	776.0	776.0
F000'	776.77	
h,k,lmax	5,22,24	5,22,24
Nref	3313	3311
Tmin,Tmax	0.971,0.989	0.781,0.862
Tmin'	0.932	

Correction method= # Reported T Limits: Tmin=0.781 Tmax=0.862
AbsCorr = MULTI-SCAN

Data completeness= 0.999 Theta(max)= 25.992

R(reflections)= 0.0610(2063) wR2(reflections)= 0.1529(3311)

S = 0.994 Npar= 245

The following ALERTS were generated. Each ALERT has the format
test-name_ALERT_alert-type_alert-level.
Click on the hyperlinks for more details of the test.

Alert level C

```
PLAT340_ALERT_3_C Low Bond Precision on  C-C Bonds ..............    0.00463 Ang.
PLAT430_ALERT_2_C Short Inter D...A Contact  O2        ..O2        .       2.88 Ang.
                                          -x,1-y,1-z  =      3_566 Check
PLAT906_ALERT_3_C Large K Value in the Analysis of Variance ......      7.173 Check
```

Alert level G

```
PLAT910_ALERT_3_G Missing FCF Reflection(s) Below Theta(Min)[Deg]=       2.04 Note
                0  1  1,
PLAT912_ALERT_4_G Missing # of FCF Reflections Above STh/L=  0.600          1 Note
PLAT933_ALERT_2_G Number of HKL-OMIT Records in Embedded .res File          1 Note
                0  1  1,
PLAT969_ALERT_5_G The 'Henn et al.' R-Factor-gap value ..........      1.550 Note
              Predicted wR2: Based on SigI**2  9.87 or SHELX Weight 15.39
PLAT978_ALERT_2_G Number C-C Bonds with Positive Residual Density.          2 Info
PLAT994_ALERT_1_G SHELXL .ins Contains no or MERG 0 Instruction ..          ! Note
```

---

0 **ALERT level A** = Most likely a serious problem - resolve or explain
0 **ALERT level B** = A potentially serious problem, consider carefully
3 **ALERT level C** = Check. Ensure it is not caused by an omission or oversight
6 **ALERT level G** = General information/check it is not something unexpected

1 ALERT type 1 CIF construction/syntax error, inconsistent or missing data
3 ALERT type 2 Indicator that the structure model may be wrong or deficient
3 ALERT type 3 Indicator that the structure quality may be low
1 ALERT type 4 Improvement, methodology, query or suggestion
1 ALERT type 5 Informative message, check

It is advisable to attempt to resolve as many as possible of the alerts in all categories. Often the minor alerts point to easily fixed oversights, errors and omissions in your CIF or refinement strategy, so attention to these fine details can be worthwhile. It is up to the individual to critically assess their own results and, if necessary, seek expert advice.

PLATON version of 15/01/2026; check.def file version of 02/01/2026

duplicate check

No duplication found

Datablock bm1615 - ellipsoid plot

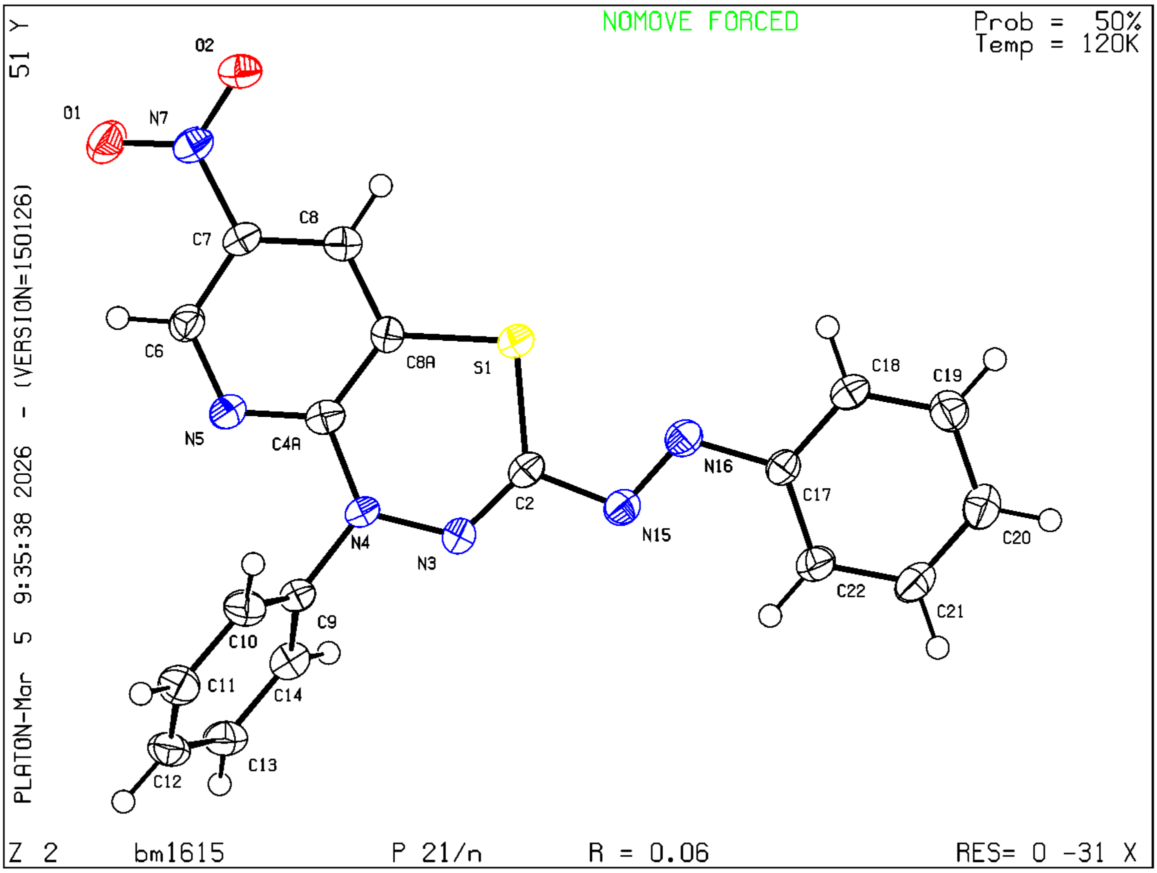

checkCIF/PLATON report

Structure factors have been supplied for datablock(s) vn961-1

THIS REPORT IS FOR GUIDANCE ONLY. IF USED AS PART OF A REVIEW PROCEDURE FOR PUBLICATION, IT SHOULD NOT REPLACE THE EXPERTISE OF AN EXPERIENCED CRYSTALLOGRAPHIC REFEREE.

No syntax errors found. CIF dictionary Interpreting this report

Datablock: vn961-1

Bond precision: C-C = 0.0016 A Wavelength=0.71073

Cell:	a=28.5254(10)	b=10.9682(4)	c=11.7688(4)
	alpha=90	beta=109.1197(9)	gamma=90
Temperature:	120 K		

	Calculated	Reported
Volume	3479.0(2)	3479.0(2)
Space group	C 2/c	C 2/c
Hall group	-C 2yc	-C 2yc
Moiety formula	C18 H10 F2 N6 O2 S	C18 H10 F2 N6 O2 S
Sum formula	C18 H10 F2 N6 O2 S	C18 H10 F2 N6 O2 S
Mr	412.38	412.38
Dx,g cm-3	1.575	1.575
Z	8	8
Mu (mm-1)	0.237	0.237
F000	1680.0	1680.0
F000'	1681.81	
h,k,lmax	40,15,16	40,15,16
Nref	5084	5067
Tmin,Tmax	0.925,0.945	0.821,0.862
Tmin'	0.925	

Correction method= # Reported T Limits: Tmin=0.821 Tmax=0.862
AbsCorr = MULTI-SCAN

```
Data completeness= 0.997            Theta(max)= 30.003

R(reflections)= 0.0315( 4185)                    wR2(reflections)=
                                                  0.0903( 5067)
S = 1.114                    Npar= 262
```

The following ALERTS were generated. Each ALERT has the format
test-name_ALERT_alert-type_alert-level.
Click on the hyperlinks for more details of the test.

Alert level C

```
PLAT911_ALERT_3_C Missing FCF Refl Between Thmin & STh/L=    0.600         16 Report
                0   2   0,   3   3   0,   5   3   0,   6   2   0,  -3   3   1,   3   1   1,
              -14   0   2,  -6   0   2,  -6   6   2,  -3   3   2,  -2   0   2,   0   0   2,
                6   0   2,  -4   0   4,   8   0   4, -21   7  10,
PLAT913_ALERT_3_C Missing # of Very Strong Reflections in FCF ....           8 Note
                3   3   0,   6   2   0,  -3   3   1,   3   1   1, -14   0   2,  -6   6   2,
               -3   3   2,  -4   0   4,
```

Alert level G

```
PLAT128_ALERT_4_G Alternate Setting for Input Space-group     C2/c       I2/a Note
PLAT432_ALERT_2_G Short Inter X...Y Contact  N3       ..C8       .       3.02 Ang.
                                                 x,1-y,-1/2+z  =      6_565 Check
PLAT910_ALERT_3_G Missing FCF Reflection(s) Below Theta(Min)[Deg]=       2.00 Note
                2   0   0,
PLAT933_ALERT_2_G Number of HKL-OMIT Records in Embedded .res File          1 Note
                2   0   0,
PLAT941_ALERT_3_G Average HKL Measurement Multiplicity ..........         4.6 Low
PLAT969_ALERT_5_G The 'Henn et al.' R-Factor-gap value ..........       4.162 Note
              Predicted wR2: Based on SigI**2  2.17 or SHELX Weight  8.10 Note
PLAT978_ALERT_2_G Number C-C Bonds with Positive Residual Density.         16 Info
```

---

0 **ALERT level A** = Most likely a serious problem - resolve or explain
0 **ALERT level B** = A potentially serious problem, consider carefully
2 **ALERT level C** = Check. Ensure it is not caused by an omission or oversight
7 **ALERT level G** = General information/check it is not something unexpected

0 ALERT type 1 CIF construction/syntax error, inconsistent or missing data
3 ALERT type 2 Indicator that the structure model may be wrong or deficient
4 ALERT type 3 Indicator that the structure quality may be low
1 ALERT type 4 Improvement, methodology, query or suggestion
1 ALERT type 5 Informative message, check

It is advisable to attempt to resolve as many as possible of the alerts in all categories. Often the minor alerts point to easily fixed oversights, errors and omissions in your CIF or refinement strategy, so attention to these fine details can be worthwhile. It is up to the individual to critically assess their own results and, if necessary, seek expert advice.

PLATON version of 23/04/2026; check.def file version of 30/03/2026

duplicate check

No duplication found

Datablock vn961-1 - ellipsoid plot

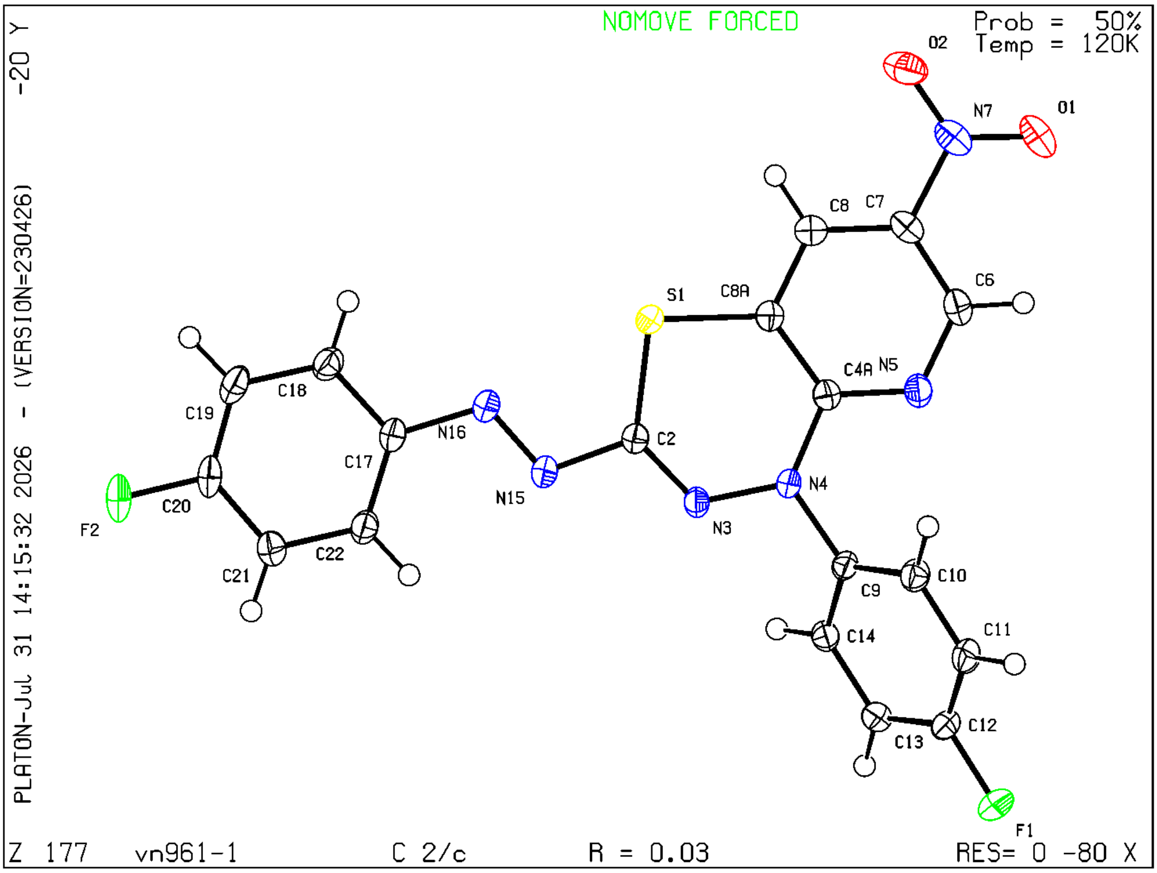

Supplement: Supplementary file 1 [file ijms-27-08886-s001.zip › SI.pdf]
