# Supplementary material for: Simple Synthesis of Fused 1,3,4-Thiadiazines Through Successive SNAr Processes
Source: Int J Mol Sci. 2026 Oct 6;27(19):8886. doi: 10.3390/ijms27198886 (PMC13665132; doi:10.3390/ijms27198886)

checkCIF/PLATON report

Structure factors have been supplied for datablock(s) vn961-1

THIS REPORT IS FOR GUIDANCE ONLY. IF USED AS PART OF A REVIEW PROCEDURE FOR PUBLICATION, IT SHOULD NOT REPLACE THE EXPERTISE OF AN EXPERIENCED CRYSTALLOGRAPHIC REFEREE.

No syntax errors found. CIF dictionary Interpreting this report

Datablock: vn961-1

```
Bond precision:   C-C = 0.0016 A          Wavelength=0.71073

Cell:            a=28.5254(10)     b=10.9682(4)        c=11.7688(4)
                 alpha=90          beta=109.1197(9)    gamma=90
Temperature:     120 K

                 Calculated                  Reported
Volume           3479.0(2)                   3479.0(2)
Space group      C 2/c                       C 2/c
Hall group       -C 2yc                      -C 2yc
Moiety formula   C18 H10 F2 N6 O2 S          C18 H10 F2 N6 O2 S
Sum formula      C18 H10 F2 N6 O2 S          C18 H10 F2 N6 O2 S
Mr               412.38                      412.38
Dx,g cm-3        1.575                       1.575
Z                8                           8
Mu (mm-1)        0.237                       0.237
F000             1680.0                      1680.0
F000'            1681.81
h,k,lmax         40,15,16                    40,15,16
Nref             5084                        5067
Tmin,Tmax        0.925,0.945                 0.821,0.862
Tmin'            0.925

Correction method= # Reported T Limits: Tmin=0.821 Tmax=0.862
AbsCorr = MULTI-SCAN
```

Data completeness= 0.997   Theta(max)= 30.003

R(reflections)= 0.0315(4185)   wR2(reflections)= 0.0903(5067)

S = 1.114   Npar= 262

The following ALERTS were generated. Each ALERT has the format
test-name_ALERT_alert-type_alert-level.
Click on the hyperlinks for more details of the test.

Alert level C

```
PLAT911_ALERT_3_C Missing FCF Refl Between Thmin & STh/L=    0.600         16 Report
                 0   2   0,    3   3   0,    5   3   0,    6   2   0,   -3   3   1,    3   1   1,
               -14   0   2,   -6   0   2,   -6   6   2,   -3   3   2,   -2   0   2,    0   0   2,
                 6   0   2,   -4   0   4,    8   0   4, -21   7 10,
PLAT913_ALERT_3_C Missing # of Very Strong Reflections in FCF ....           8 Note
                 3   3   0,    6   2   0,   -3   3   1,    3   1   1, -14   0   2,   -6   6   2,
                -3   3   2,   -4   0   4,
```

Alert level G

```
PLAT128_ALERT_4_G Alternate Setting for Input Space-group     C2/c       I2/a Note
PLAT432_ALERT_2_G Short Inter X...Y Contact  N3       ..C8       .       3.02 Ang.
                                              x,1-y,-1/2+z  =      6_565 Check
PLAT910_ALERT_3_G Missing FCF Reflection(s) Below Theta(Min)[Deg]=       2.00 Note
                 2   0   0,
PLAT933_ALERT_2_G Number of HKL-OMIT Records in Embedded .res File          1 Note
                 2   0   0,
PLAT941_ALERT_3_G Average HKL Measurement Multiplicity ..........         4.6 Low
PLAT969_ALERT_5_G The 'Henn et al.' R-Factor-gap value ..........       4.162 Note
               Predicted wR2: Based on SigI**2  2.17 or SHELX Weight  8.10 Note
PLAT978_ALERT_2_G Number C-C Bonds with Positive Residual Density.         16 Info
```

---

0 **ALERT level A** = Most likely a serious problem - resolve or explain
0 **ALERT level B** = A potentially serious problem, consider carefully
2 **ALERT level C** = Check. Ensure it is not caused by an omission or oversight
7 **ALERT level G** = General information/check it is not something unexpected

0 ALERT type 1 CIF construction/syntax error, inconsistent or missing data
3 ALERT type 2 Indicator that the structure model may be wrong or deficient
4 ALERT type 3 Indicator that the structure quality may be low
1 ALERT type 4 Improvement, methodology, query or suggestion
1 ALERT type 5 Informative message, check

It is advisable to attempt to resolve as many as possible of the alerts in all categories. Often the minor alerts point to easily fixed oversights, errors and omissions in your CIF or refinement strategy, so attention to these fine details can be worthwhile. It is up to the individual to critically assess their own results and, if necessary, seek expert advice.

PLATON version of 23/04/2026; check.def file version of 30/03/2026

duplicate check

No duplication found

Datablock vn961-1 - ellipsoid plot

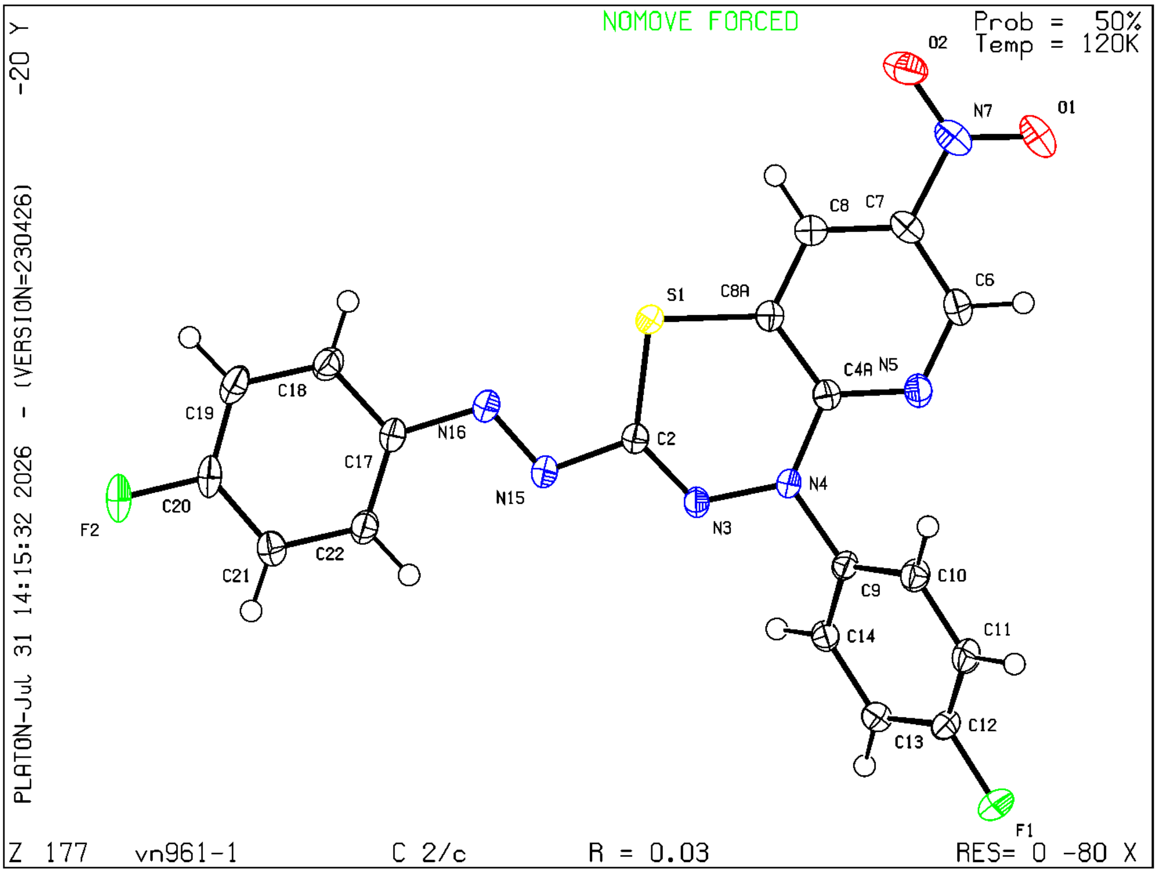

Supplement: Supplementary file 1 [file ijms-27-08886-s001.zip › vn961-1.checkcif.pdf]
